# Herbal pair Huangqin-Baishao: mechanisms underlying inflammatory bowel disease by combined system pharmacology and cell experiment approach

**DOI:** 10.1186/s12906-020-03068-2

**Published:** 2020-09-25

**Authors:** Xiaoqi Huang, Zhiwei Chen, Minyao Li, Yaomin Zhang, Shijie Xu, Haiyang Huang, Xiaoli Wu, Xuebao Zheng

**Affiliations:** 1 Mathematical Engineering Academy of Chinese Medicine, Guangdong Provincial Key Laboratory of New Chinese Medicinal Development and Research, Guangzhou University of Chinese Medicine, 232# Wai Huan East Road, Guangzhou Higher Education Mega Center, Guangzhou, 510006 China; 2 Dongguan Mathematical Engineering Academy of Chinese Medicine, Guangzhou University of Chinese Medicine, Dongguan, 523808 China; 3 School of Biomedical and Pharmaceutical Sciences, Guangdong University of Technology, 100# Wai Huan West Road, Guangzhou Higher Education Mega Center, Guangzhou, 510006 China; 4 Dongguan Songshan Lake Yi Dao TCM Clinic, Dongguan, 523808 China

**Keywords:** HQ-BS herbal pair, Inflammatory bowel disease, Synergic mechanism, System pharmacology, THP-1 cells

## Abstract

**Background:**

Inflammatory bowel disease (IBD) is a severe digestive system condition, characterized by chronic and relapsing inflammation of the gastrointestinal tract. Scutellaria baicalensis Georgi (Huangqin, HQ) and *Paeonia lactiflora* Pall (Baishao, BS) from a typical herbal synergic pair in traditional Chinese medicine (TCM) for IBD treatments. However, the mechanisms of action for the synergy are still unclear. Therefore, this paper aimed to predict the anti-IBD targets and the main active ingredients of the HQ-BS herbal pair.

**Methods:**

A systems pharmacology approach was used to identify the bioactive compounds and to delineate the molecular targets and potential pathways of HQ-BS herbal pair. Then, the characteristics of the candidates were analyzed according to their oral bioavailability and drug-likeness indices. Finally, gene enrichment analysis with DAVID Bioinformatics Resources was performed to identify the potential pathways associated with the candidate targets.

**Results:**

The results showed that, a total of 38 active compounds were obtained from HQ-BS herbal pair, and 54 targets associated with IBD were identified. Gene Ontology and pathway enrichment analysis yielded the top 20 significant results with 54 targets. Furthermore, the integrated IBD pathway revealed that the HQ-BS herbal pair probably acted in patients with IBD through multiple mechanisms of regulation of the nitric oxide biosynthetic process and anti-inflammatory effects. In addition, cell experiments were carried out to verify that the HQ-BS herbal pair and their Q-markers could attenuate the levels of nitric oxide (NO), prostaglandin E_2_ (PGE_2_), inducible nitric oxide synthase (iNOS) and cyclooxygenase-2 (COX-2) in lipopolysaccharide (LPS)-stimulated THP-1-derived macrophage inflammation. In particular, the crude materials exerted a much better anti-inflammatory effect than their Q-markers, which might be due to their synergistic effect.

**Conclusion:**

This study provides novel insight into the molecular pathways involved in the mechanisms of the HQ-BS herbal pair acting on IBD.

## Background

Inflammatory bowel disease (IBD) including Crohn’s disease (CD) and ulcerative colitis (UC), is a chronic and recurring intestinal inflammatory disorder. Although a number of commercial drugs have been used to treat IBD in the clinic, a lack of sustained efficacy and side effects hinder their application. It has been reported that one-third of UC patients and two-thirds of CD patients need to undergo surgery eventually [1, 2]. TCM has been widely used to treat different kinds of diseases in China for a long time. TCM usually consist of different herbal medicines mixed together in specific quantities to form a formula according to the composition theory “Monarch, Minister, Assistant, and Guide”. In the formula, Monarch means the main drug for the etiology and symptoms; Minister means promoting the therapeutic effect produced by Monarch; Assistant means treating the accompanying symptom or eliminating the side effects of the Monarch; and Guide means guiding the formula to the disease area or reconciling its efficacy [3–5]. To improve the quality of life, it is vital to develop nontoxic and sustained efficacy medicines to treat IBD.

Studies have reported various beneficial effects of TCM and herbal extracts in treating IBD including anti-bacterial, anti-inflammatory, and anti-cancer activities [6, 7]. Among them, HuangQin decoction (HQD) is a famous formula recorded in the Treatise on Exogenous Febrile Disease written by Zhongjing Zhang. HQD has been used to treat gastrointestinal disease such as diarrhea, abdominal spasms, vomiting, and nausea for a long time in China [8]. HQD consists of four ingredients: the roots of *Scutellaria baicalensis Georgi* (Huangqin, HQ), *Paeonia lactiflora Pall* (Baishao, BS), *Glycyrrhiza uralensis Fisch* (Gancao, GC), and the fruit of *Ziziphus jujuba Mill* (Dazao, DZ) [9]. HQD has been proven to ameliorate intestinal damage [10]. According to the composition theory, HQ is the Monarch, and BS is the Minister in this formula. HQ-BS is composed of a typical herbal synergic pair and is widely used for colitis treatments. PF2405, a standardized fraction of Scutellaria baicalensis, shows prominent anti-inflammatory action in ameliorating colitis in vitro and in vivo [11]. Moreover, Dai et al. used a system pharmacology method and experiments to discover and prove Q-markers of HQD in the role of attenuating IBD, and nine constituents (paeoniflorin, baicalin, scutellarein, liquiritigenin, norwogonin, baicalein, glycyrrhizic acid, wogonin, and oroxylin A) were found to be Q-markers of HQD. Moreover, all of these compounds are the main constituents of HQ-BS [9]. Baicalin was proved to exert a remarkable anti-inflammatory effect on colitis induced by dextran sodium sulfate (DSS), and the mechanistic investigation revealed that it may be regulated through the caudal-type homeobox 2 (CDX2/pregnane X receptor (PXR) pathway [12, 13]. Furthermore, Luo and colleagues provided evidence that baicalin attenuated colitis induced by trinitro-benzene-sulfonic acid (TNBS) via inhibiting the toll like receptor-4 (TLR4)/myeloid differential protein-88 (MyD88) signaling cascade and inactivating the NOD-like receptor 3 (NLRP3) inflammasome [14]. Zhang et al. proved that paeoniflorin abrogated colitis induced by DSS via reducing the expression of TLR4 and inhibiting the activation of nuclear factor kappa-B (NF-κB) and the mitogen-activated protein kinase (MAPK) pathway [15]. Gu et al. showed that paeoniflorin exerted anti-inflammatory and anti-apoptosis actions in ulcerative colitis by suppressing the MAPK/NF-κB pathway in mice [16].

**Fig. 1:**
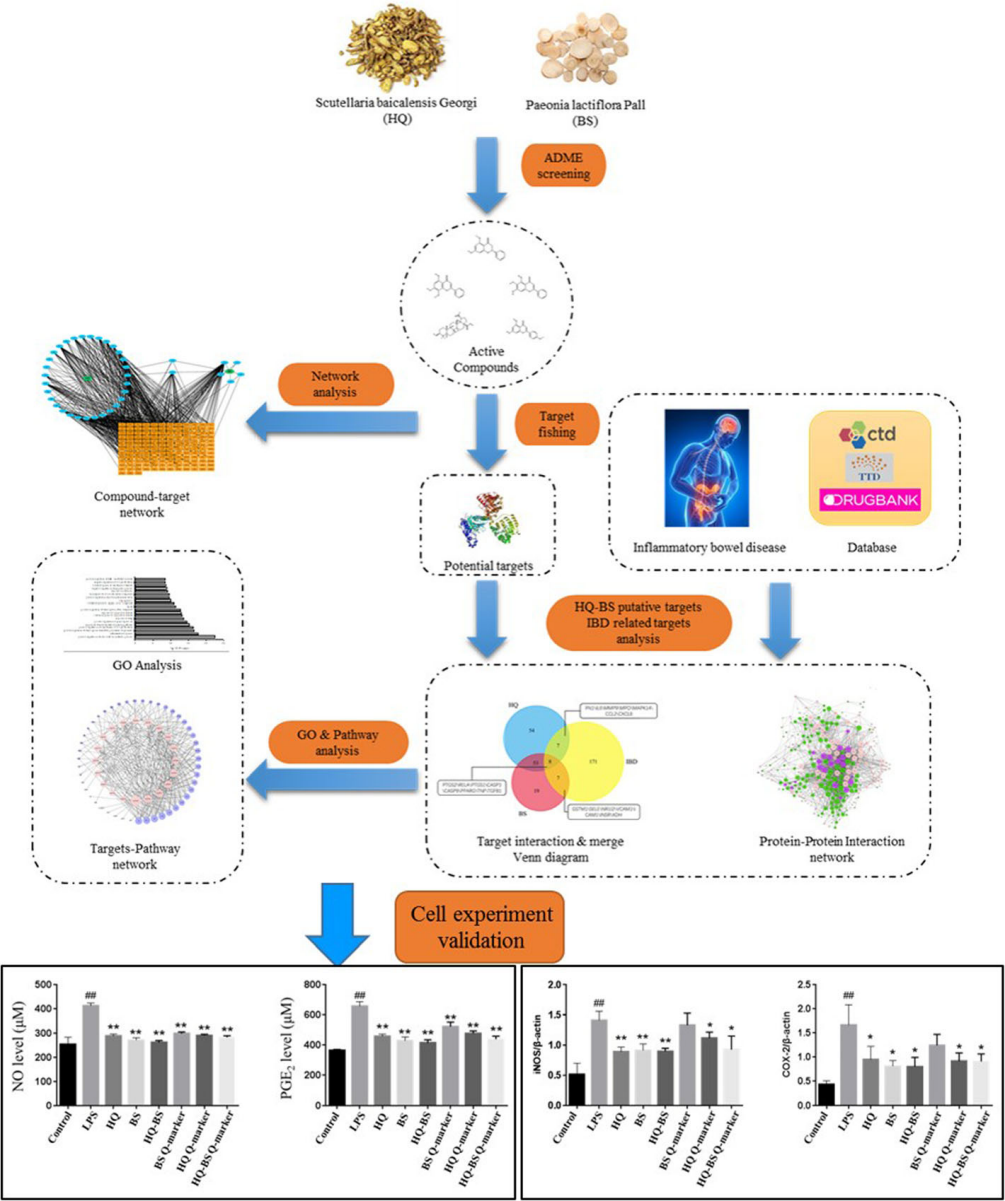
Systems pharmacology approach workflow for elucidating the mechanisms of actions for the synergy of the HQ-BS herbal pair IBD

Although HQD and its bioactive components exert significant effects in attenuating IBD, the specific bioactive molecules in HQD and the underlying mechanisms of action remain unknown. Currently, system pharmacology has successfully been widely used to investigate the role and the mechanism of TCM [17–23]. Thus, in the current study, we use a system pharmacology approach combined with cell experiments (the protocol is shown in Fig. 1) to investigate the active constituents, targets and underlying pathways of the HQ-BS herbal pair.

## Materials and methods

### Data preparation

The chemical compound data and the targets of HQ and BS were obtained from the Traditional Chinese Medicine Systems Pharmacology Database (TCMSP, http://sm.nwsuaf.edu.cn/lsp/tcmsp.php.) [24] and were manually supplemented. Target information of the ingredients was obtained from DrugBank (www.drugbank.ca) [25]. Anti-IBD targets were derived from the Therapeutic Targets Database (TTD, http://bidd.nus.edu.sg/group/ttd/ttd.asp) [26], Comparative Toxicogenomics Database (CTD, http://ctdbase.org/) [27] and PharmGKB (http://www.pharmgkb.org) [28]. Protein-protein interaction (PPI) data were derived from the STRING database (http://string-db.org) [29]. Putative targets were enriched in the Kyoto Encyclopedia of Genes and Genomes (KEGG, http://www.kegg.jp/ or http://www.genome.jp/kegg/) [30]. All of the targets were mapped to the database UniProt (http://www.uniprot.org) [31] to construct the networks of Herb-Compound-Target (H-C-T).

### Network construction and network analysis

Networks were constructed using the data about the active constituents, corresponding targets, and pathway information. Moreover, we used an open software package project named Cytoscape v1.1 Core (http://www.cytoscape.org/) [32] to construct all of the visualized network graphs.

### Gene ontology and pathway enrichment analysis

Gene Ontology (GO) enrichment analysis for the putative targets of the HQ and BS herbal pair was conducted using the Database for Annotation, Visualization and Integrated Discovery (DAVID, https://david.ncifcrf.gov) [33]. DAVID was also applied to perform pathway enrichment analysis for the same gene targets to identify the potential synergistic actions of HQ and BS against IBD. Enriched GO terms and pathways were defined as those with false discovery rate (FDR) adjusted *P* values < 0.05.

### Cell culture

THP-1 cells (human monocytes derived from a patient with acute monocytic leukemia) were cultured with RPMI 1640 medium containing 10% fetal bovine serum and 1% penicillin-streptomycin at 37 °C under a humidified atmosphere with 5% (*v/v*) CO_2._

#### Chemicals and reagents

LPS (L4391, a purity no less than 99%) was purchased from Sigma (St. Louis, USA,); HQ and BS crude materials were obtained from Kangmei Pharmaceutical Co. LTD (Jieyang, China) and authenticated by Prof. Ziren Su (Guangzhou University of Chinese Medicine); paeoniflorin (CAS: 23180–57-6), baicalin (CAS: 21967–41-9), baicalein (CAS: 352000–07-8), wogonin (CAS: 632–85-9), and oroxylin A (CAS: 23180–57-6) were purchased from Dalian Meilun Biotechnology Co., Ltd. (Dalian, China), the purities are no less than 98%; NO assay kit (A013–2-1) was purchased from Nanjing Jiancheng Bioengineering Institute (Nanjing China); PGE_2_ (CSB-E07966m), iNOS (CSB-E08326m) and COX-2 (CSB-E12910m) ELISA kits were supplied from CUSABIO (Wuhan China). The iNOS anti-body (AF0199) and COX-2 anti-body (AF7003) were purchased from Affinity Biosciences Inc. (Cincinnati, USA).

### Drug groups design

LPS-stimulated THP-1-derived macrophage inflammation was used as a cell model to assess the Q-markers and crude medicines from the HQ-BS herbal pair. We designed the groups as follows: HQ crude material (51 μg/ml), BS crude material (34 μg/ml), HQ-BS crude material pair (85 μg/ml), BS Q-markers (paeoniflorin 3.218 μg/ml), HQ Q-markers (baicalin 17.026 μg/ml + baicalein 0.59 μg/ml + wogonin 0.316 μg/ml + oroxylin A 0.1 μg/ml and the HQ-BS herbal pair Q-markers (paeoniflorin 3.218 μg/ml + baicalin 17.026 μg/ml + baicalein 0.59 μg/ml + wogonin 0.316 μg/ml + oroxylin A 0.1 μg/ml). The crude materials were extracted with distilled water and concentrated. The Q-markers were dissolved in DMSO and then diluted with culture medium to become the Q-markers of the HQ-BS decoction. The incubation concentration of the materials and the Q-markers were calculated from 400 mg/ml of HQD.

### NO and PGE_2_ release assay

THP-1 cells were seeded in 96-well plates at 10,000 per well for 12 h. Subsequently, the drugs mentioned above were added to the cells and they were incubated for 12 h. Then, the cells were subjected to 1 mg/ml LPS for 24 h, except for the vehicle control group. Finally, the culture medium was collected for the NO and PGE_2_ assays using commercial ELISA kits according to the manufacturer’s instructions.

### iNOS and COX-2 assays

THP-1 cells were seeded in 6-well plates at 100,000 per well for 12 h. Subsequently, the drugs mentioned above were added to the cells and they were incubated for 12 h. Then, the cells were subjected to 1 mg/ml LPS for 24 h, except for the vehicle control group. Finally, the cells were collected for iNOS and COX-2 assay using Western blot. The cells were lysed in chilled lysis buffer containing protease and phosphatase inhibitors for 20 min on ice. Afterward, they were centrifuged at 14000 g and 4 °C for 10 min and the protein concentration of the supernatant was measured using a BCA Protein Assay Kit according to the protocol provided by the manufacturer. The protein samples were separated on SDS-PAGE gels and then transferred onto PVDF membranes. The membranes were blocked with 5% skim milk in TBST. Then, the membranes were incubated with iNOS and COX-2 primary antibodies at 4 °C overnight followed by incubation with the secondary antibodies for 1 h at room temperature. Finally, the bands were detected by an ECL reagent using a Western blotting detection system.

### Statistical analysis

All data are expressed as the mean ± standard deviation (SD) and analyzed by one-way analysis of variance (ANOVA) followed by a least-significant difference test. *p*-values of < 0.05 or <0.01 were regarded as statistically significant. The statistical analysis was performed by SPSS 20.0 (SPSS Inc., NY, USA).

## Results

### Ingredient comparisons in the HQ-BS herbal pair

**Fig. 2:**
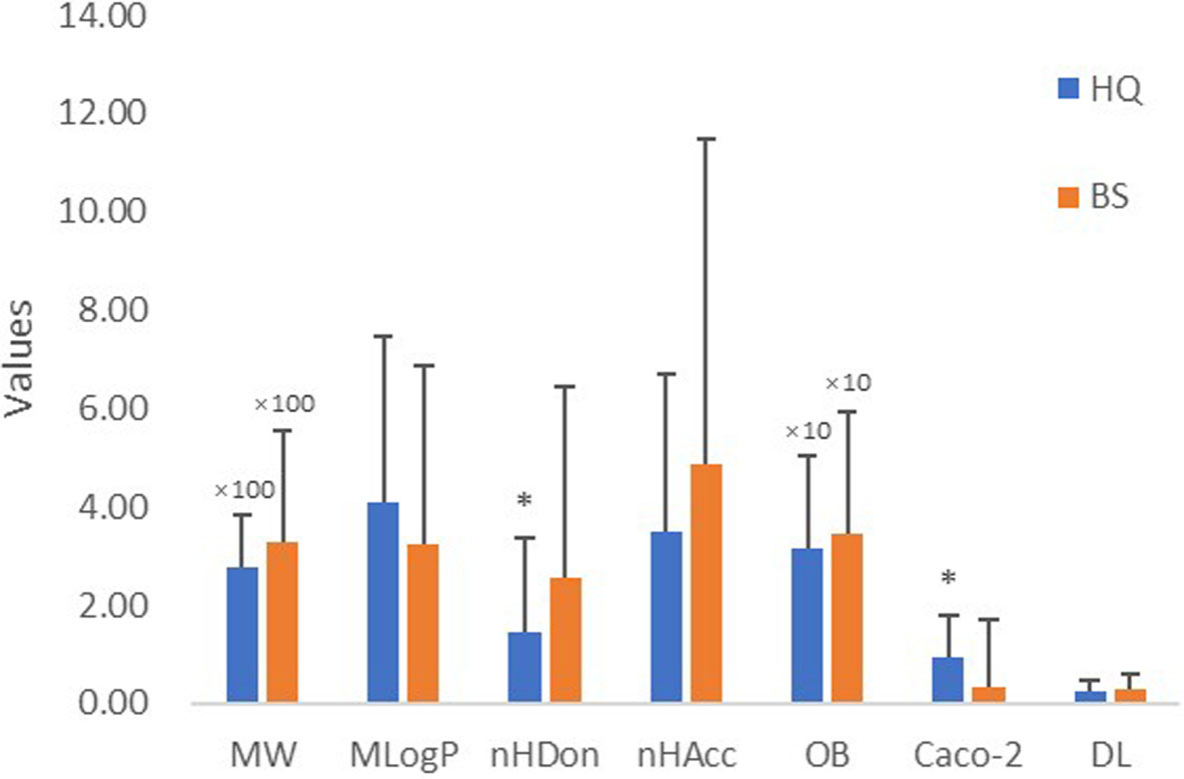
Properties comparison of ingredients from HQ-BS herbal pair. *p < 0.05 by two tailed t-test (vs. BS)

The ingredients in the HQ-BS pair were retrieved from the TCMSP. All of the constituents from the HQ-BS herbal pair were used to construct a compound library. A total of 215 constituents were obtained for HQ (143) and BS (85), respectively. The main ingredients of HQ are flavonoids and alkaloids, and the major constituents in BS are triterpenoids. We evaluated the molecular diversities of all the constituents from the HQ-BS herbal pair based on the following properties as shown in Fig. 2).

1. The average molecular weight (MW) of each ingredient from HQ and BS was calculated, and there was no significant difference (*p* = 0.06) between HQ (277.74) and BS (326.75).
2. The Moriguchi octanol-water partition coeff. (logP) (MLogP) of each ingredient from HQ and BS was calculated, and there was no significant difference (*p* = 0.07) between HQ (4.10) and BS (3.22). This result suggests that hydrotropic constituents are the main ingredients in both HQ and BS.
3. The number of donor atoms for the H-bonds (nHDon) of each ingredient from HQ and BS was calculated, and the average nHDon number of BS constituents (2.55) was much higher than that of the HQ constituents (1.44) (*p* = 0.02).
4. The number of acceptor atoms for H-bonds (nHAcc) of each ingredient from HQ and BS was calculated and there was no significant difference (*p* = 0.07) between HQ (3.48) and BS (4.86).
5. The average oral bioavailability (OB) value of the constituents of HQ (31.42) and BS (34.43) was without significant difference (*p* = 0.33).
6. The Caco-2 permeability Caco-2 of each ingredient from HQ and BS was calculated, and the average nHDon number of BS constituents (0.328) was much lower than that of the HQ constituents (0.94) (*p* = 3.58 × 10^− 4^).
7. For drug-likeness (DL) analysis, the average DL index of the constituents of HQ and BS were 0.23 and 0.29, respectively, which displays no significant difference (*p* = 0.11).

Despite the constituents of HQ and BS being diverse, based on the analysis above, we also found that the majority of them met the Lipinski’s rule of five. Apart from the nHDon, HQ was similar to BS in other properties of the chemical components. These similarities indicate that the constituents of HQ and BS share similar chemo-physical properties and that the ingredients from HQ and BS possessed similar DL. These results also indicate that the compounds from the HQ-BS herbal pair had similar pharmacokinetic properties.

### Active ingredients in the HQ-BS herbal pair

Although the HQ-BS herbal pair contains a large number of bioactive compounds, only a few that exert desirable pharmacodynamic and pharmacokinetic properties contribute to the therapeutic actions of this herbal pair. In the current study, we screened the active constituents from the HQ-BS herbal pair from the following absorption, distribution, metabolism, and excretion (ADME) parameters, including OB, Caco-2 and DL. Some constituents were also chosen for their effective bioactivities without satisfying all of these criteria. As a result, a total of 38 active compounds (33 active compounds from HQ and 7 active compounds from BS, and among them, two compounds found in both HQ and BS) were chosen from the 215 compounds of this herbal pair (shown in Supplementary Table 1).

### Targets of the HQ-BS herbal pair that are anti-IBD

**Fig. 3:**
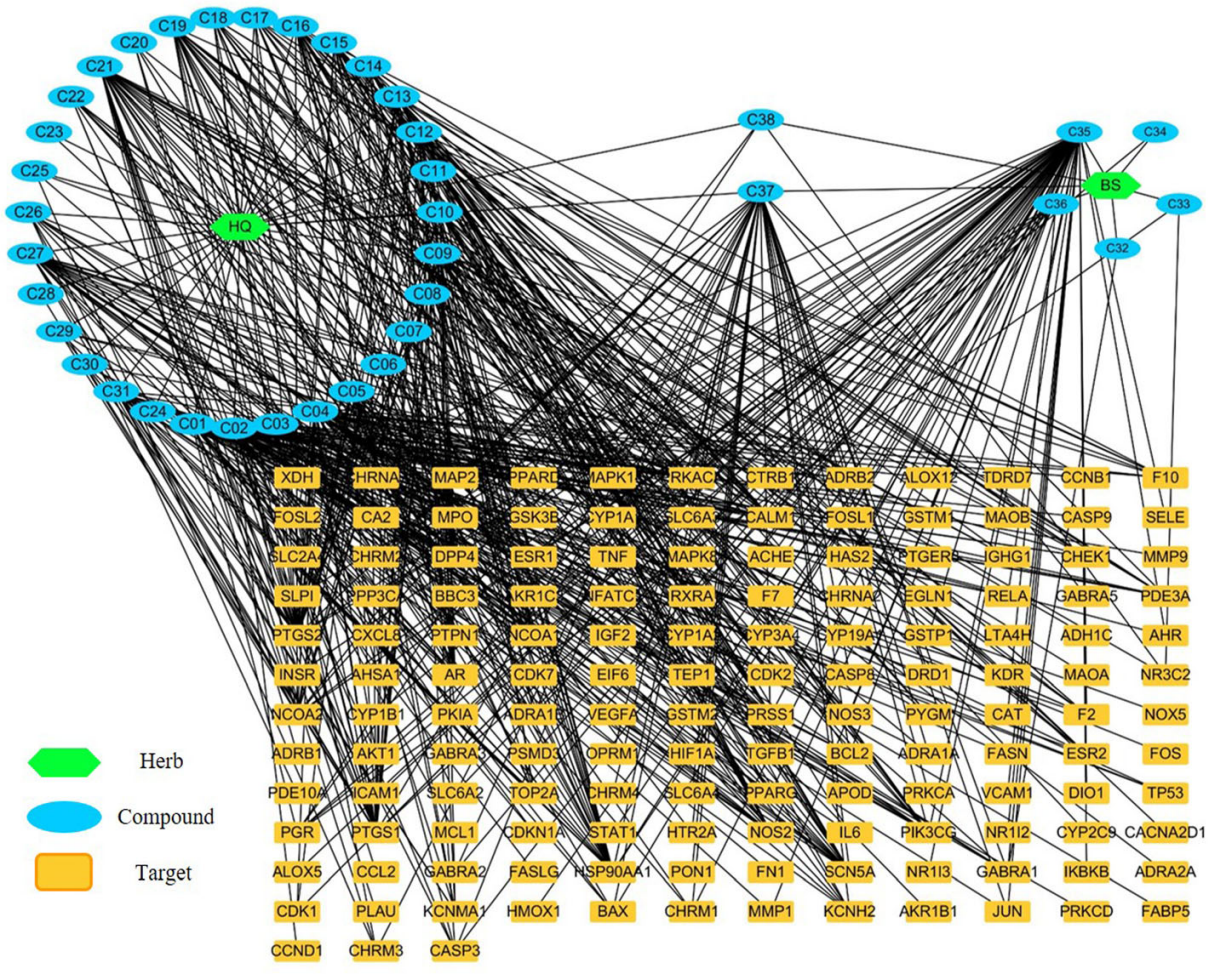
Herb-Compound-Target (H-C-T) Network. The green nodes represent herbs, the blue nodes represent compounds, and the orange nodes represent HQ-BS putative targets, respectively

To investigate the synergic mechanism of the HQ-BS herbal pair for IBD, the parameters of the active compounds of HQ and BS were obtained from the TCMSP and the target information was collected from DrugBank. Herb Ingredients’ Target (HIT, http://lifecenter.sgst.cn/hit/) [34] was used for specific targets validated by the experiments and the SysDT model [35] was used for the targets without experimental verification. As shown in Supplementary Table 2 and Fig. 3, 147 putative targets that belong to 38 active ingredients of the HQ-BS pair were collected. All of the above targets were mapped to the database UniProt. Subsequently, we constructed the networks of H-C-T using these data. Finally, we calculated the contribution index of all the active constituents based on the networks.

### Interactions between the HQ-BS putative targets and the anti-IBD targets

**Fig. 4:**
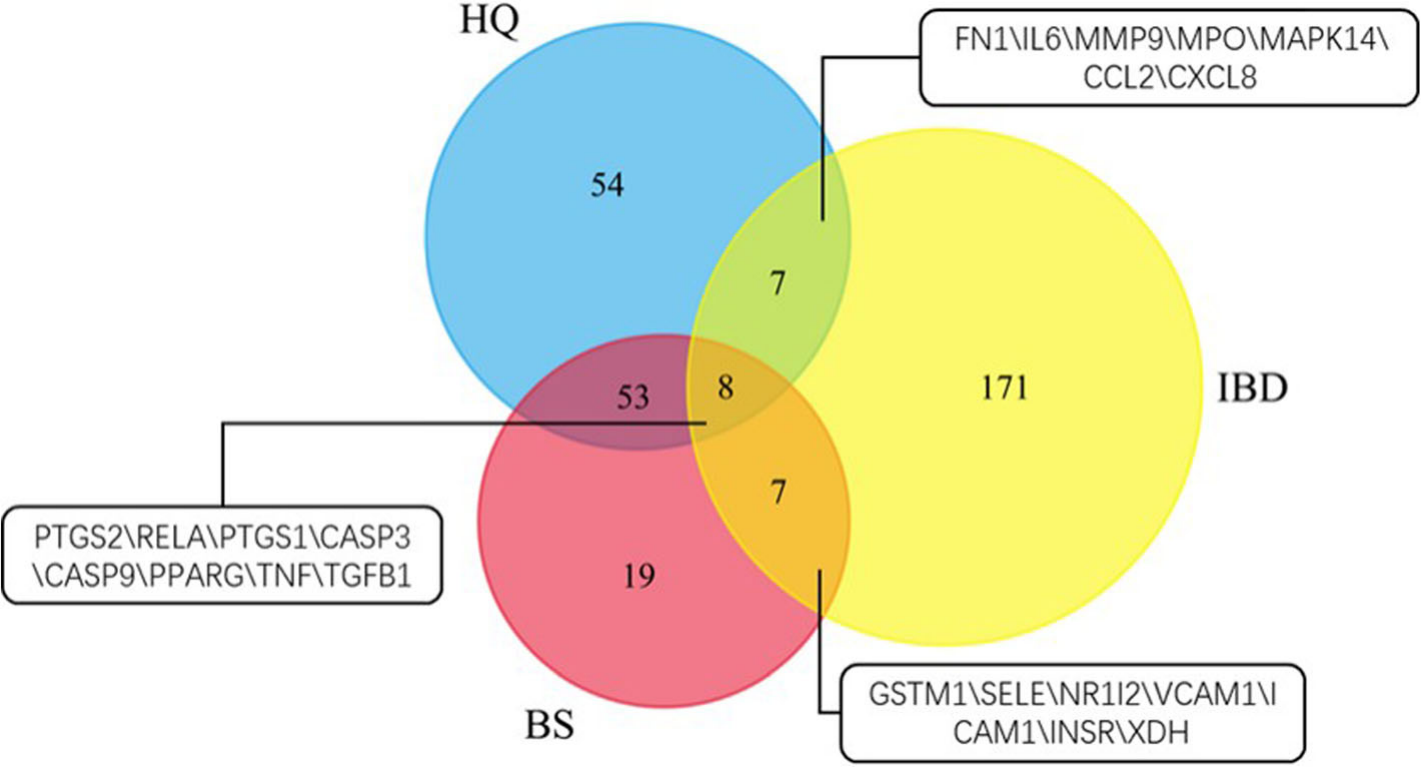
Direct putative anti-IBD targets of HQ-BS herbal pair. Venn diagram for putative targets of HQ-BS herbal pair and known anti-IBD targets. The targets in each intersection set were also listed

Targets for IBD were derived from the TTD, CTD and PharmGKB by using the keywords “inflammatory bowel disease (IBD)”, “Crohn’s disease” and “ulcerative colitis”. As shown in Fig. 4, the three target sets, anti-IBD targets (yellow), putative targets for HQ (blue), and putative targets for BS (red), had intersections containing targets. Among the intersected targets, 8 targets were at the intersections of three targets sets, 7 targets were at the intersections of the blue and yellow sets, and 7 targets were at the intersections of the red and yellow sets. The 22 targets were considered to be the direct putative HQ-BS herbal pair anti-IBD targets.

**Fig. 5:**
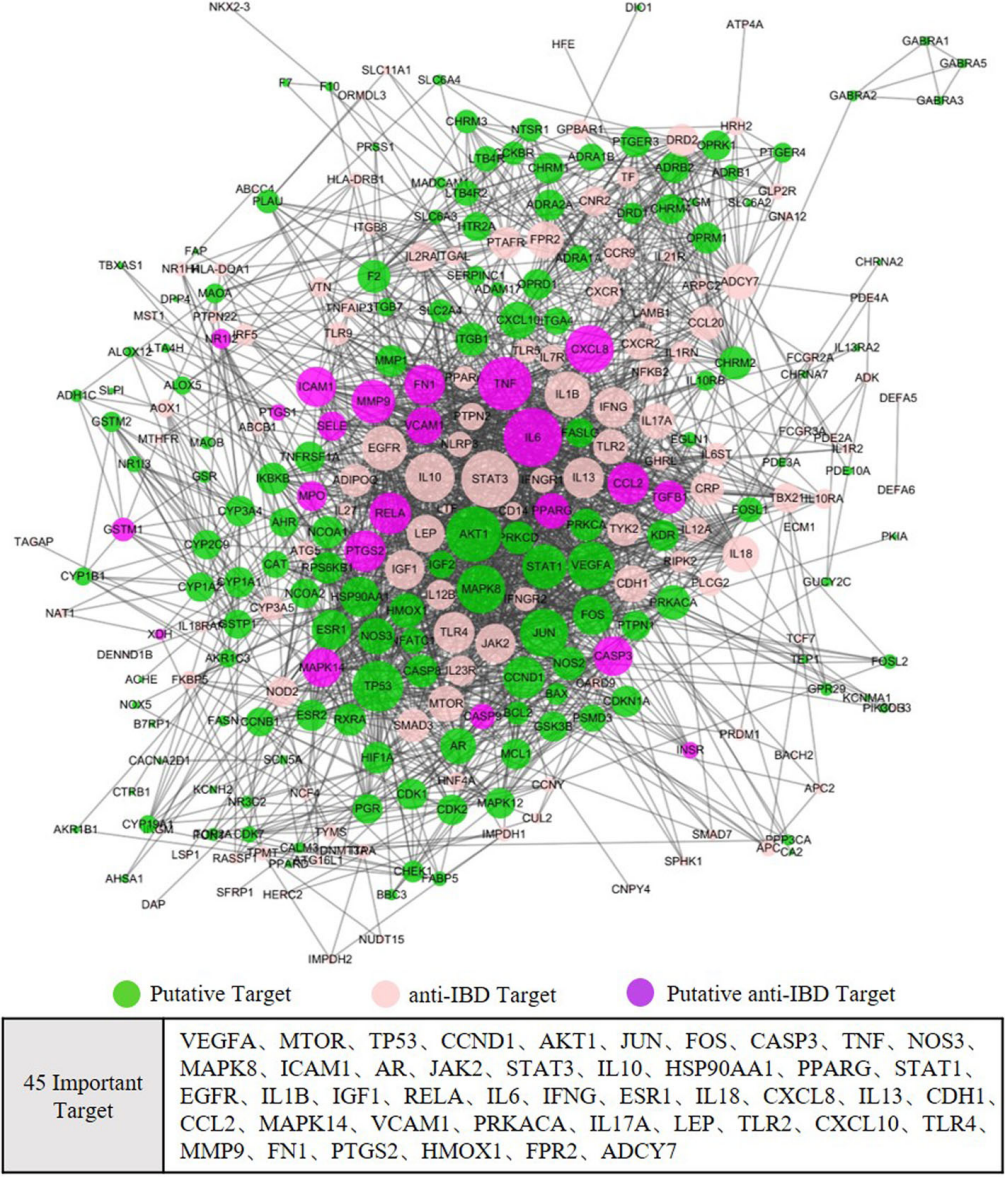
Protein-protein interaction (PPI) network of anti-IBD targets and proteins that indirectly interact with the targets. The PPI network of anti-IBD protein targets (The size of the circle represents the degree of a node); A list of 45 crucial proteins which interact with the anti-IBD targets

Next, we constructed a PPI network using the 193 IBD targets and the 147 HQ-BS putative targets. The PPI was ranked among four minimum required interaction scores (low, the score is 0.150; medium, 0.4; high, 0.7; and highest, 0.9). We selected the high scoring (scores > 0.7) PPIs to construct the PPI network. Our analysis obtained a connected network of 319 proteins and 2093 interactions. From them, 66 were anti-IBD targets, 385 were HQ-BS putative targets, and in the intersection lay 26 HQ-BS against IBD targets. The specific interactions are depicted in Fig. 5a. With a hub node [36] defined as a node with a degree higher than 2-fold of the average degree of all the nodes in the networks (the average degree of all the nodes in the network was 13 in our study), 45 major hub nodes were identified (Fig. 5b).

### Functions of the HQ-BS herbal pair for IBD by GO enrichment

**Fig. 6:**
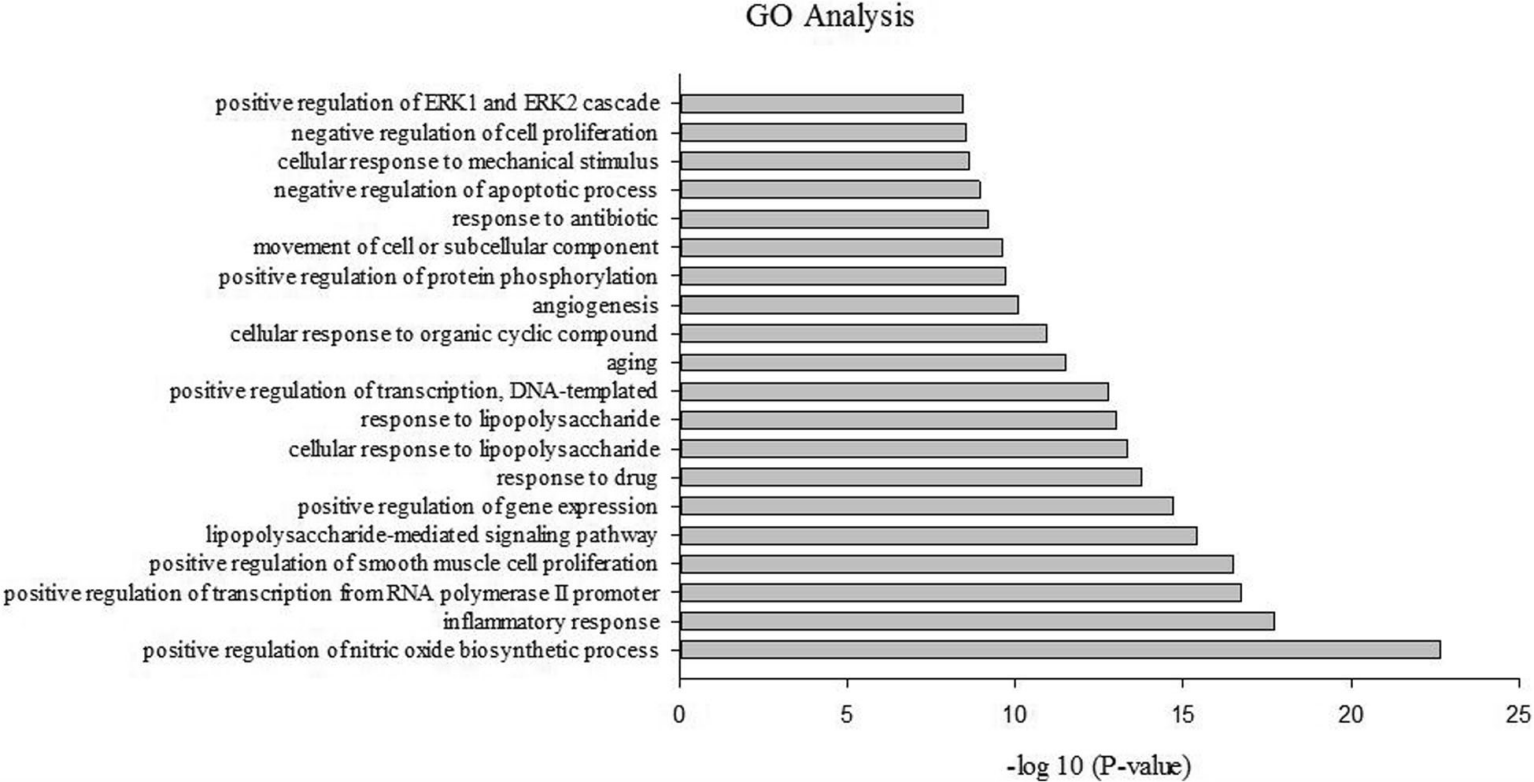
the top 20 biological processes by GO enrichment analysis of therapeutic targets. The 푥-axis represents the enrichment scores of these terms (*p* < 0.05), and the 푦-axis represents significantly enriched BP categories in GO relative to the targets

As shown in Table 1 and Fig. 6, GO enrichment analysis of the 54 putative targets of HQ and BS (combining the 22 IBD related targets with 45 important targets) resulted in the top 20 biological processes (BP) with FDR corrected *p* < 0.05. The results indicate that they are involved in signal transduction, regulation, and response processes, such as positive regulation of nitric oxide biosynthetic process (25.93%), inflammatory response (37.04%), lipopolysaccharide-mediated signaling pathway (18.52%), and cellular response to lipopolysaccharide (22.22%). Consistent with prior publications, HQ and BS are targeting different proteins in the nitric oxide biosynthetic process pathway.

**Table 1:** The top 20 biological processes enriched by the 54 putative targets

Term	Count of Proteins	%	*p* -Value
positive regulation of nitric oxide biosynthetic process	14	25.93	2.12 × 10^−23^
inflammatory response	20	37.04	1.89 × 10^−18^
positive regulation of transcription from RNA polymerase II promoter	26	48.15	1.90 × 10^−17^
positive regulation of smooth muscle cell proliferation	12	22.22	3.09 × 10^−17^
lipopolysaccharide-mediated signaling pathway	10	18.52	4.01 × 10^−16^
positive regulation of gene expression	16	29.63	1.99 × 10^−15^
response to drug	16	29.63	1.76 × 10^−14^
cellular response to lipopolysaccharide	12	22.22	4.69 × 10^−14^
response to lipopolysaccharide	13	24.07	9.47 × 10^−14^
positive regulation of transcription, DNA-templated	18	33.33	1.69 × 10^−13^
aging	12	22.22	3.15 × 10^−12^
cellular response to organic cyclic compound	9	16.67	1.11 × 10^−11^
angiogenesis	12	22.22	8.27 × 10^−11^
positive regulation of protein phosphorylation	10	18.52	2.04 × 10^−10^
movement of cell or subcellular component	9	16.67	2.49 × 10^−10^
response to antibiotic	7	12.96	6.28 × 10^−10^
negative regulation of apoptotic process	14	25.93	1.12 × 10^−9^
cellular response to mechanical stimulus	8	14.81	2.36 × 10^−9^
negative regulation of cell proliferation	13	24.07	2.79 × 10^−9^
positive regulation of ERK1 and ERK2 cascade	10	18.52	3.53 × 10^−9^

### Pathway analysis to explore the underlying mechanisms of the HQ-BS herbal pair

**Fig. 7:**
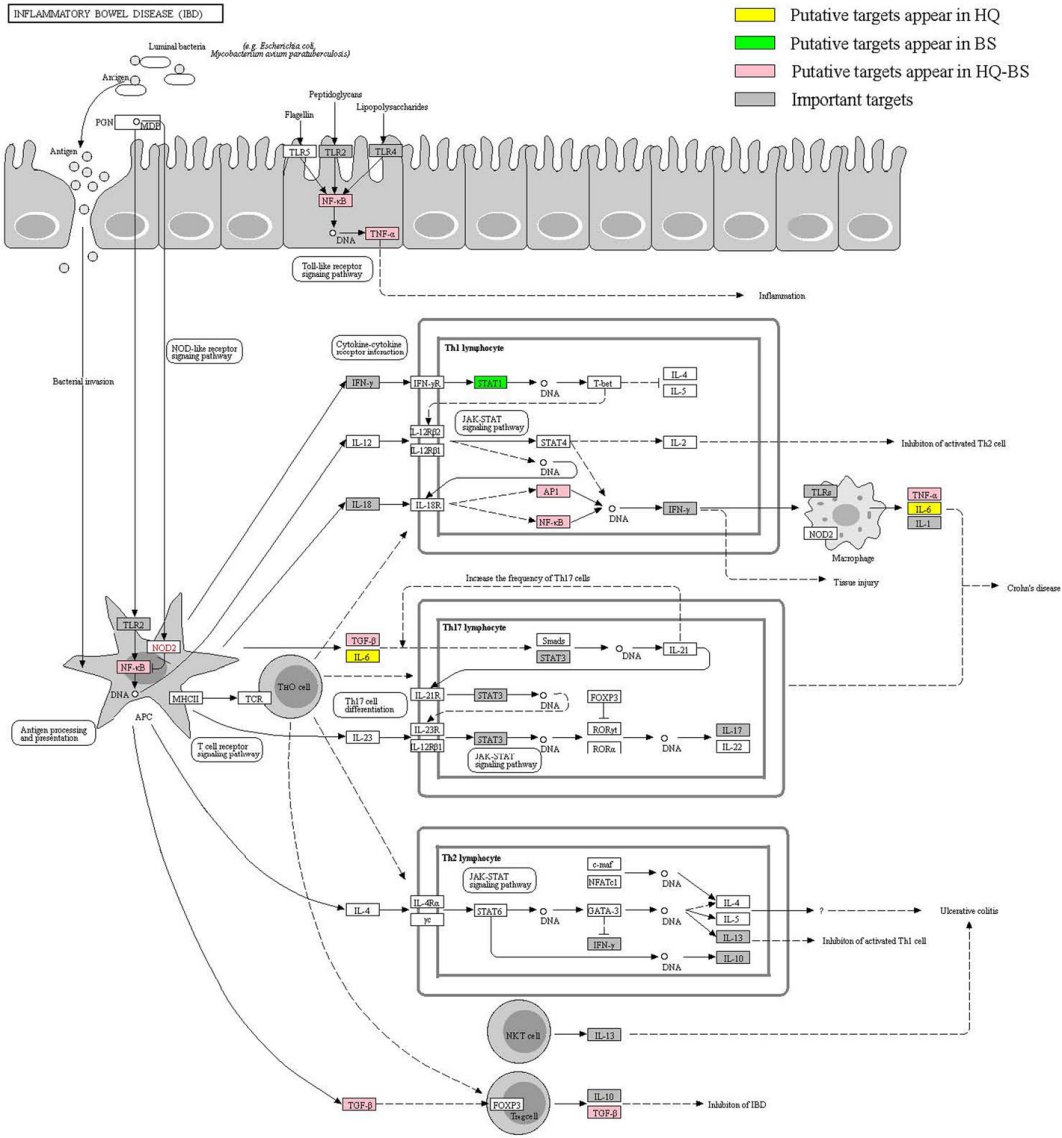
Distribution of partial targets of HQ-BS on the IBD pathway. Target-Pathway (P-T) Network

**Fig. 8:**
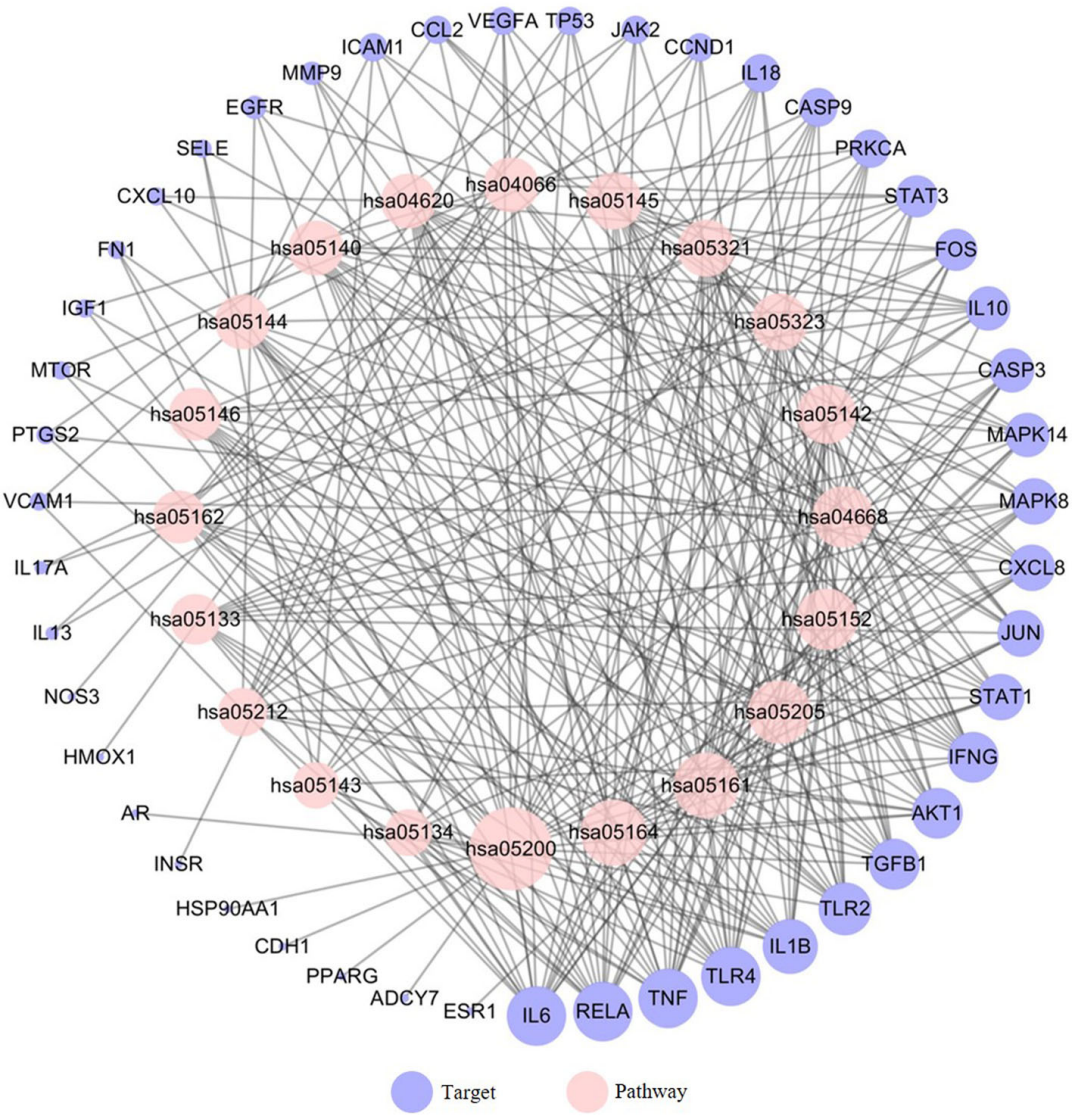
Target-Pathway network for HQ-BS anti-IBD. The pink and blue nodes represent the pathway and targets, respectively, and the edges represent the interactions among them

The KEGG pathway enrichment analysis suggested that the 54 putative targets of HQ and BS were enriched with FDR corrected *p* < 0.05. As shown in Supplementary Table 3, the top 20 pathways associated with the HQ and BS herbal pair were obtained. Consistent with the previous publications, the tumor necrosis factor (TNF) signaling pathway, Toll-like receptor signaling pathway, and the IBD pathway had a strong correlation with the HQ-BS herbal pair against IBD. Furthermore, we investigated the distribution of partial targets of HQ-BS on the IBD pathway (shown in Fig. 7). After analysis by the Target-Pathway (T-P) network, 67 nodes (20 pathways and 47 proteins) and 303 interactions were obtained (shown in Fig. 8).

### The HQ-BS herbal pair attenuated NO and PGE_2_ release

**Fig. 9:**
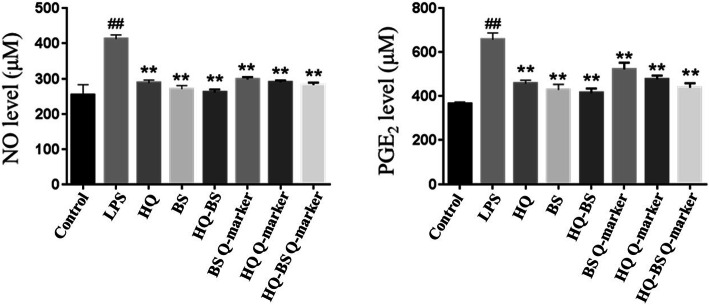
HQ, BS, HQ-BS herbal pair and their Q-markers attenuated the levels of NO and PGE_2_. Data were expressed as the mean ± SD (*n* = 8). ^##^*p* < 0.01 compared with control group; ^*^p < 0.05, ^**^p < 0.01 compared with LPS group

The results showed that an LPS insult induced a significant release of NO and PGE_2_ compared with the control cells. However, HQ, BS and their active components treatment could significantly attenuate the levels of NO and PGE_2_ (as shown in Fig. 9). Especially, the crude materials of HQ, BS and HQ-BS exerted much stronger activities in attenuating the release of NO and PGE_2_ compared with the Q-markers of HQ, BS and HQ-BS. These results indicate that the excellent anti-inflammatory effects of HQ-BS might be due to the synergistic effect of all their components.

### The HQ-BS herbal pair reduced the levels of iNOS and COX-2

**Fig. 10:**
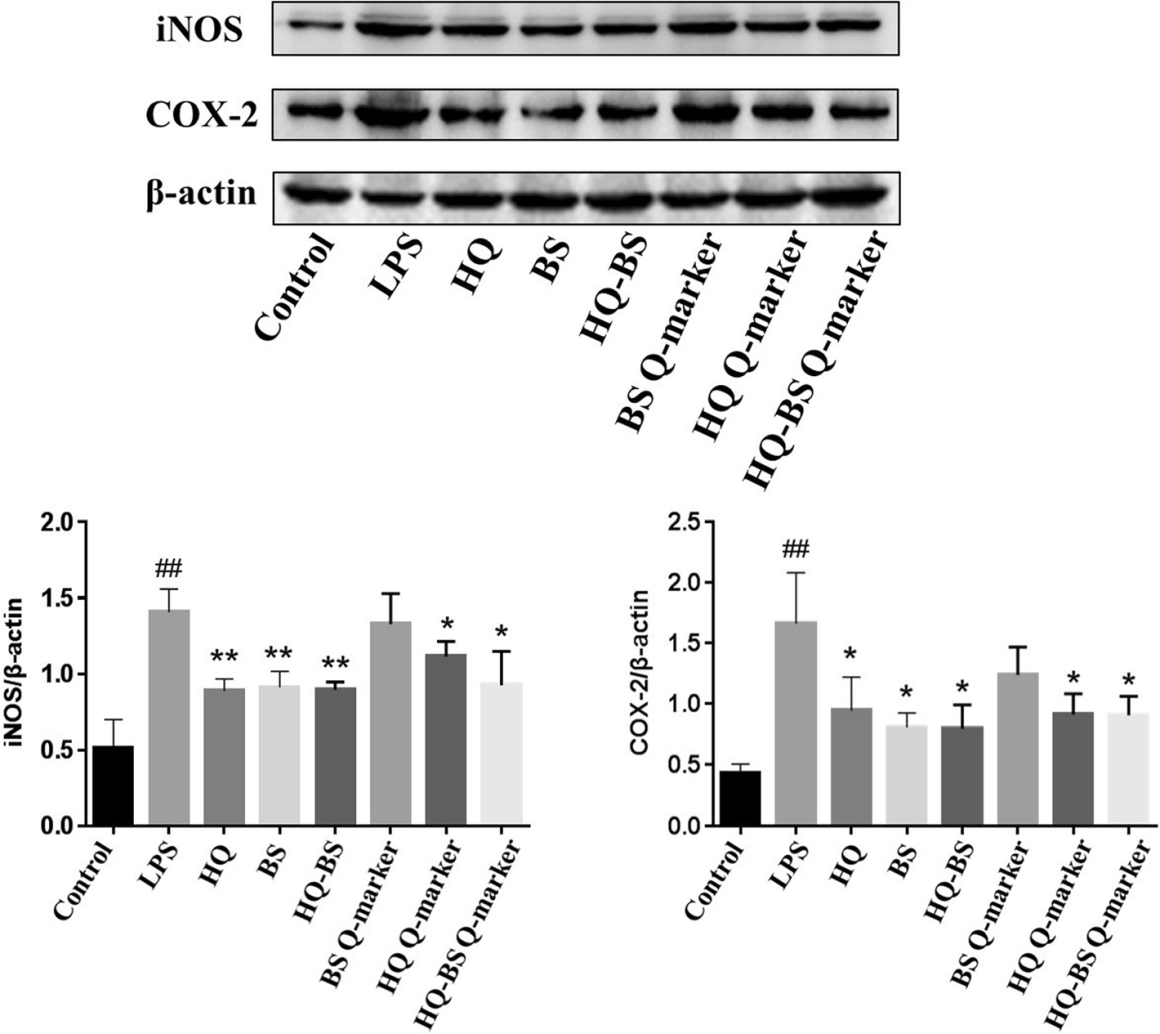
HQ, BS, HQ-BS herbal pair and their Q-markers attenuated the levels of NO and PGE_2_. Data were expressed as the mean ± SD (*n* = 3). ^##^*p* < 0.01 compared with control group; ^*^p < 0.05, ^**^p < 0.01 compared with LPS group

Western blot results showed that the levels of iNOS and COX-2 increased sharply after LPS stimulation compared with the control cells. However, HQ, BS and their active components treatment could significantly reduce the levels of NO and PGE_2_ (as shown in Fig. 10). Similar to the NO and PGE_2_ assay result, the crude materials of HQ, BS and HQ-BS could remarkably attenuate the inflammatory response remarkably than their Q-markers. The Western blot results further confirmed that their synergistic effect plays an important role in the anti-inflammatory effects of HQ-BS.

## Discussion

In the current study, we used a systems pharmacology approach to investigate the bioactive ingredients and significant pathways of the HQ-BS herbal pair. Then, we performed in vitro cell experiments to verify the network pharmacology prediction results.

A total of 215 compounds of the HQ and BS herbal pair were obtained from the TCMSP database, and 38 of them were chosen for further investigation following the drug-likeness analysis. Among the 38 compounds, 31 compounds were present in HQ, 5 compounds were present in BS, and 2 compounds were present in both HQ and BS.

By analyzing these 38 bioactive compounds of HQ and BS, we found that alkaloids and flavonoids were the primary active moieties of HQ, and triterpenoids were the main active constituents of BS. In HQ, flavonoid constituents, such as baicalin, wogonin, acacetin, skullcapflavone, carthamin, dihydrooroxylin and oroxylin A are known to be anti-inflammatory agents [37]. Especially, baicalin was proven to exert excellent effects in ameliorating colitis through polarization of macrophages to an M2 phenotype, inflammation inhibition, and the suppression of oxidant stress and apoptosis [37–41]. The alkaloid composition, coptisine, was previously reported to attenuate intestinal damage [42, 43]. In BS, the triterpenoids composition, such as paeoniflorin, was reported to attenuate intestinal damage by inhibiting apoptosis and the NF-kappa B and TLR4 pathways [15, 16]. By analyzing 54 putative anti-IBD targets of HQ and BS, we found that the targets were mainly from the following two anti-IBD target classes: (1) apoptosis-related targets (such as heat shock proteins, apoptosis regulators, and caspases) and (2) 13 inflammation-related targets (such as prostaglandin, nitric oxide synthase, interleukin-6, and tumor necrosis factor) [32]. Prostaglandin plays a pivotal role in allergic inflammation. Moreover, reducing the level of prostaglandin exerts a protective effect against IBD and may represent a future safe treatment for IBD [44–46]. Furthermore, treatment with natural products could attenuate colitis by downregulating the level of prostaglandin. Nitric oxide synthase [47–49], interleukin-6 [50], and tumor necrosis factor [51] also revealed the same effect as prostaglandin in the regulation of IBD. Additionally, the apoptosis-related targets, such as bax, bcl-2, caspase-3 and caspase-9, also have crucial role in the pathogenesis of IBD [52]. By analyzing the top 20 KEGG pathways of the 54 putative targets, we found that positive regulation of the nitric oxide biosynthetic process was the most correlated pathway. The nitric oxide biosynthetic process plays an essential role in the pathobiology of IBD. In IBD, iNOS induces the generation of NO, which leads to the release of pro-inflammatory cytokines, such as TNF-α, interleukin-1β (IL-1β) and interleukin-6 (IL-6). The accumulation of NO and pro-inflammatory cytokines in the intestinal epithelial cells triggers intestinal inflammation in IBD [53]. Cellular responses to lipopolysaccharide, inflammatory response and the TNF signaling pathway are also associated with the mechanism of HQ-BS against IBD.

The system pharmacology prediction results above suggest that cellular response to lipopolysaccharide, inflammatory response and the TNF signaling pathway are also associated with the mechanism of HQ-BS against IBD. Moreover, the HQ-BS herbal pair is predicted to target the nitric oxide biosynthetic process and inflammation-related targets (such as prostaglandin, nitric oxide synthase, interleukin-6, and tumor necrosis factor). Hence, to verify whether the HQ-BS herbal pair attenuates IBD symptom by targeting the inflammation response, we performed in vitro cell experiments to confirm their effects. The LPS-induced inflammation model in THP-1 cells is an accepted model to evaluate the actions of drugs on inflammation. A previous report demonstrated that paeoniflorin from BS is a Q-marker of HQD, while baicalin, baicalein, wogonin, and oroxylin A from BS are Q-markers of HQD [9]. Therefore, we tested the effect of HQ, BS, the HQ-BS herbal pair and their Q-markers against the LPS-induced inflammatory response in the THP-1 cells. The results showed that HQ, BS, the HQ-BS herbal pair and their Q-markers could suppress NO and PGE_2_ production, as well as iNOS and COX-2 protein expression. These cell-based results suggest that the synergistic effect plays an important role in the anti-inflammatory effects of HQ-BS.

Based upon the above prediction and experimental results, the underlying mechanism of the HQ-BS herbal pair against IBD may be associated with the regulation of the nitric oxide biosynthetic process and inflammatory cytokines release.

## Conclusion

The mechanism of the HQ-BS herbal pair in IBD involves multiple ingredients, targets, and pathways. Combining the prediction and cell experimental results, the therapeutic effects of the HQ-BS herbal pair in IBD may be dependent on the regulation of the proteins and pathways related to the nitric oxide biosynthetic process and the inflammatory response by inhibiting NO synthesis and inflammatory cytokine release. The systems pharmacology approach combined with cell experimental methods as applied in our study provided an alternative strategy for the comprehensive understanding of the mechanisms of the HQ-BS herbal pair in IBD. In the future, we would like to validate the therapeutic effects of the HQ-BS herbal pair in IBD using a mouse model. Furthermore, we will investigate the mechanism whether by the HQ-BS herbal pair attenuates IBD through affecting the nitric oxide biosynthetic process and the inflammatory response.

## Supplementary information

**Additional file 1: Table S1.** Compounds of *Scutellaria baicalensis* (Huangqi) and *Paeonia lactiflora* (Baishao). **Table S2.** Targets of HQ-BS pair active compounds. **Table S3.** The top 20 KEGG pathways of 54 putative targets generated by DAVID.

**Additional file 2.** Original blot images.

## Data Availability

The dataset used during the current study is stored in a secured research data server at Guangzhou University of Chinese Medicine. The datasets used are available from the corresponding author upon reasonable request.

## Abbreviations

IBD: Inflammatory bowel disease
HQ: Huangqin
BS: Baishao
TCM: Traditional Chinese medicine
NO: Nitric oxide
PGE_2_: Prostaglandin E_2_
iNOS: Inducible nitric oxide synthase
COX-2: Cyclooxygenase-2
LPS: Lipopolysaccharide
CD: Crohn’s disease
UC: Ulcerative colitis
HQD: HuangQin decoction
GC: Gancao
DZ: Dazao
DSS: Dextran sodium sulfate
CDX2: Caudal-type homeobox 2
PXR: Pregnane X receptor
TNBS: Trinitro-benzene-sulfonic acid
TLR4: Toll like receptor-4
MyD88: Myeloid differential protein-88
NLRP3: NOD-like receptor 3
NF-κB: Nuclear factor kappa-B
MAPK: Mitogen-activated protein kinase
TCMSP: Traditional Chinese Medicine Systems Pharmacology Database
TTD: Therapeutic Targets Database
CTD: Comparative Toxicogenomics Database
PPI: Protein-protein interaction
KEGG: Kyoto Encyclopedia of Genes and Genomes
H-C-T: Herb-Compound-Target
GO: Gene Ontology
DAVID: Database for Annotation, Visualization and Integrated Discovery
FDR: False discovery rate
SD: Standard deviation
ANOVA: Analysis of variance
MW: Molecular weight
MLogP: Moriguchi octanol-water partition coeff. (logP)
nHDon: Number of donor atoms for the H-bonds
nHAcc: Number of acceptor atoms for H-bonds
OB: Oral bioavailability
DL: Drug-likeness
ADME: Absorption, distribution, metabolism, and excretion
HIT: Herb Ingredients’ Target
BP: Biological processes
TNF: Tumor necrosis factor
T-P: Target-Pathway
IL-1β: Interleukin-1β
IL-6: Interleukin-6
