# Supplementary material for: Herbal pair Huangqin-Baishao: mechanisms underlying inflammatory bowel disease by combined system pharmacology and cell experiment approach
Source: BMC Complement Med Ther. 2020 Sep 25;20:292. doi: 10.1186/s12906-020-03068-2 (PMC7523401; doi:10.1186/s12906-020-03068-2)
Supplement: Supplementary file 1 — Additional file 1: Table S1. Compounds of Scutellaria baicalensis (Huangqi) and Paeonia lactiflora (Baishao). Table S2. Targets of HQ-BS pair active compounds. Table S3. The top 20 KEGG pathways of 54 putative targets generated by DAVID. [file 12906_2020_3068_MOESM1_ESM.docx]

Supplementary table 1 Compounds of *Scutellaria baicalensis* (Huangqi) and *Paeonia lactiflora* (Baishao)

| No | Chemical Name | Structure | OB(%) | Caco-2 | DL | Herb |
| --- | --- | --- | --- | --- | --- | --- |
| C01 | Acacetin | 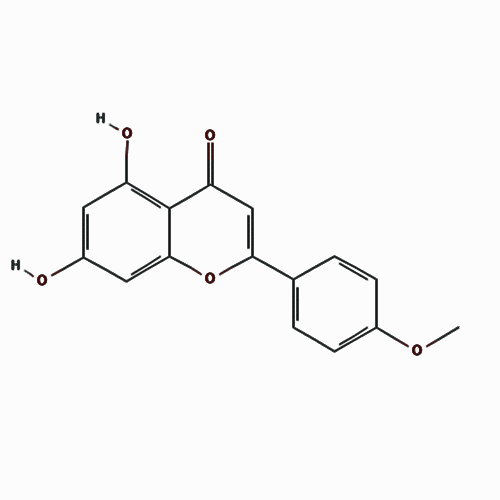 | 34.97 | 0.67 | 0.24 | *Scutellaria baicalensis* |
| C02 | Wogonin | 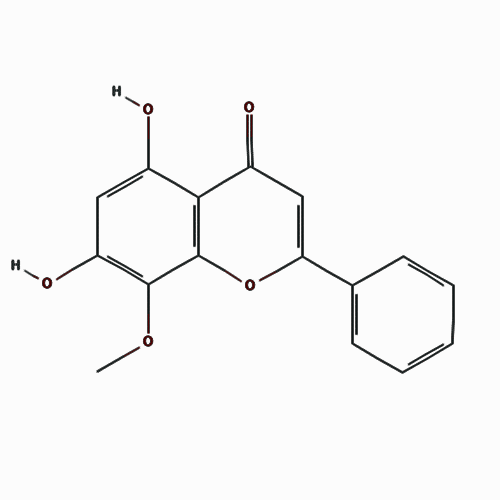 | 30.68 | 0.79 | 0.23 | *Scutellaria baicalensis* |
| C03 | (2R)-7-Hydroxy-5-methoxy-2-phenyl-2,3-dihydrochromen-4-one | 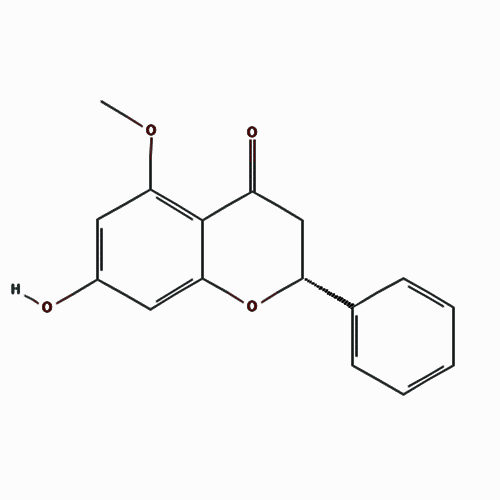 | 55.23 | 0.87 | 0.20 | *Scutellaria baicalensis* |
| C04 | Baicalein | 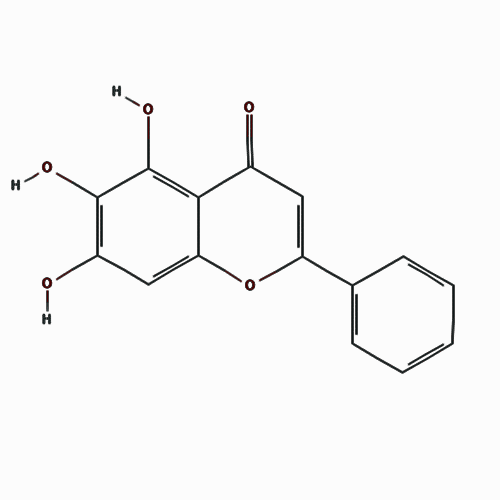 | 33.52 | 0.63 | 0.21 | *Scutellaria baicalensis* |
| C05 | 5,8,2'-Trihydroxy-7-methoxyflavone | 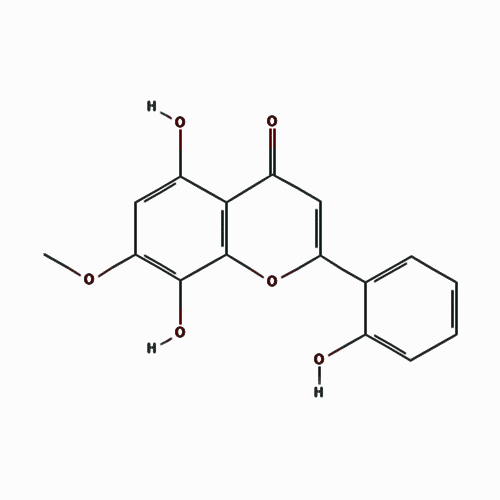 | 37.01 | 0.76 | 0.27 | *Scutellaria baicalensis* |
| C06 | 5,7,3',6'-Tetrahydroxy-6,8,2'-trimethoxyflavone | 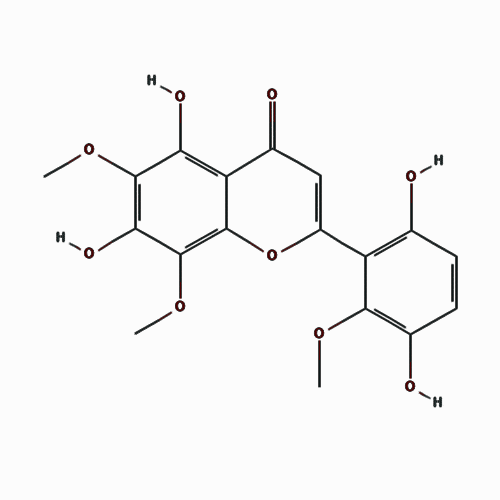 | 33.82 | 0.35 | 0.45 | *Scutellaria baicalensis* |
| C07 | Carthamidin | 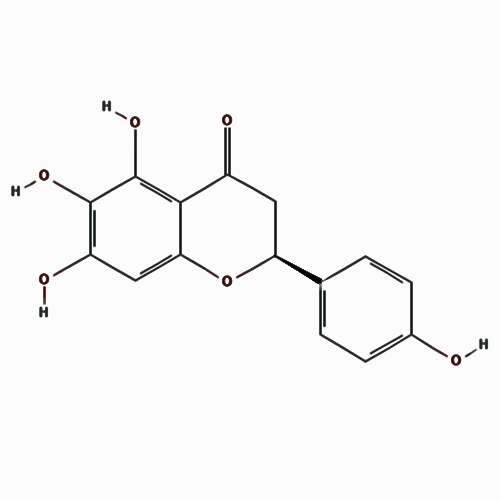 | 41.15 | 0.16 | 0.24 | *Scutellaria baicalensis* |
| C08 | Dihydrobaicalein | 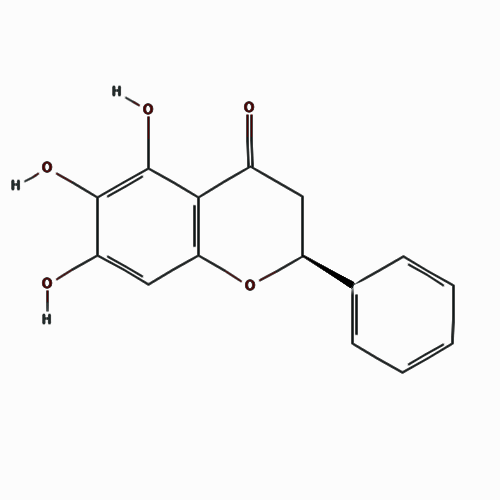 | 40.04 | 0.56 | 0.21 | *Scutellaria baicalensis* |
| C09 | Eriodyctiol (Flavanone) | 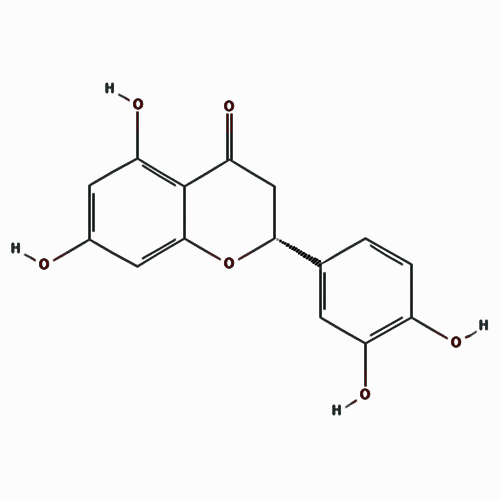 | 41.35 | 0.05 | 0.24 | *Scutellaria baicalensis* |
| C10 | Salvigenin | 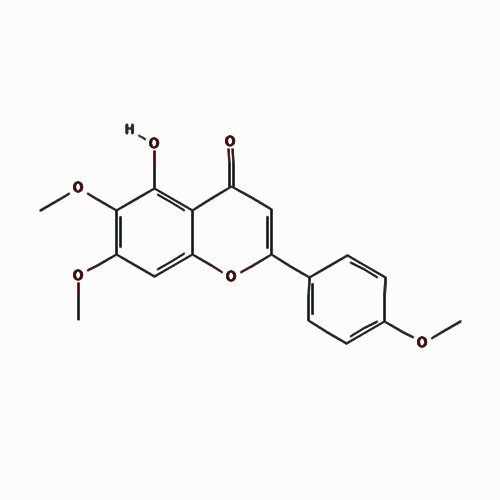 | 49.07 | 0.86 | 0.33 | *Scutellaria baicalensis* |
| C11 | Viscidulin II | 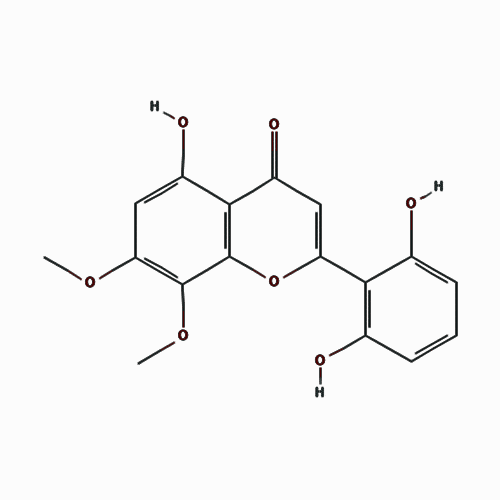 | 45.05 | 0.48 | 0.33 | *Scutellaria baicalensis* |
| C12 | 5,7,2',6'-Tetrahydroxyflavone | 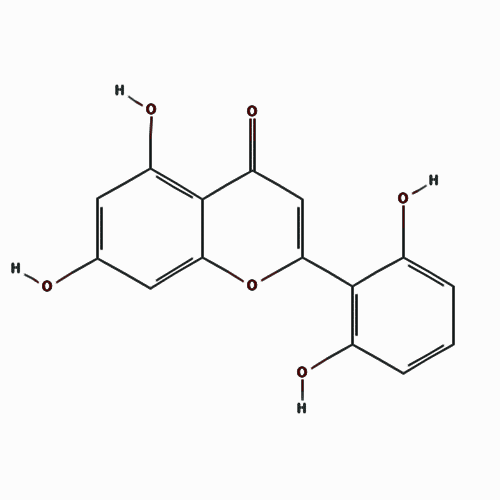 | 37.01 | 0.18 | 0.24 | *Scutellaria baicalensis* |
| C13 | Skullcapflavone II | 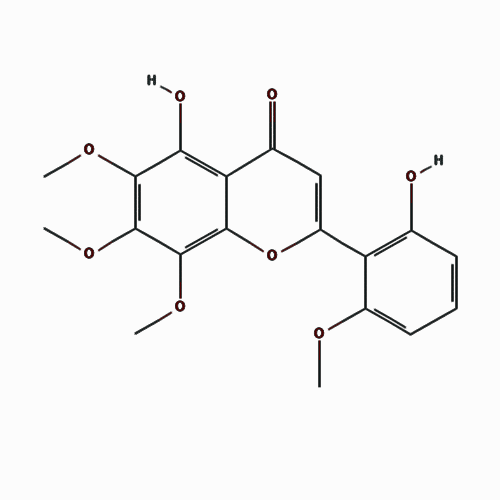 | 69.51 | 0.68 | 0.44 | *Scutellaria baicalensis* |
| C14 | Oroxylin A | 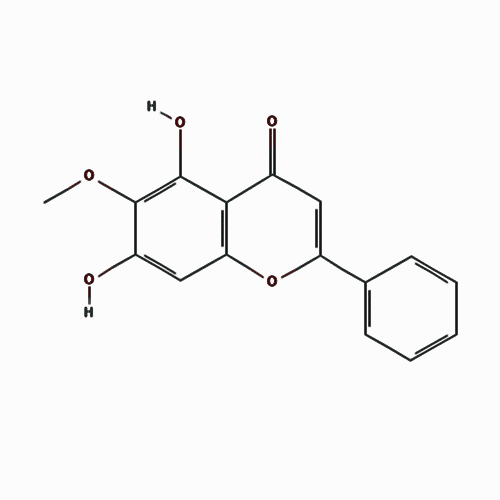 | 41.37 | 0.76 | 0.23 | *Scutellaria baicalensis* |
| C15 | Skullcapflavone I | 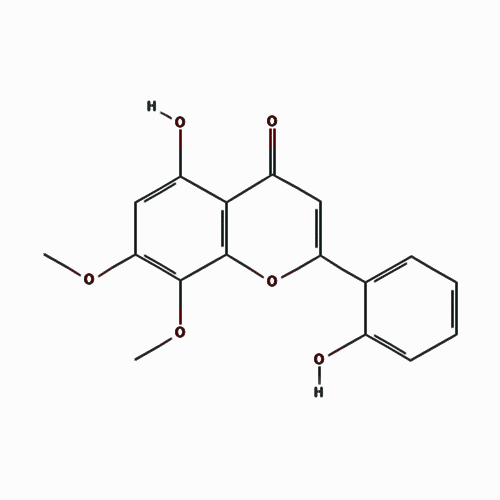 | 76.26 | 0.84 | 0.29 | *Scutellaria baicalensis* |
| C16 | 5,7,4'-Trihydroxy-8-methoxyflavone | 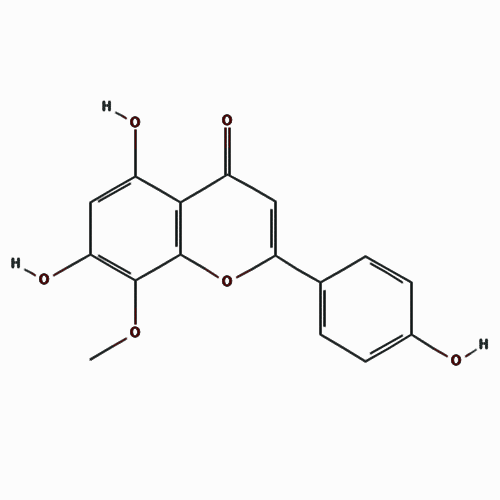 | 36.56 | 0.46 | 0.27 | *Scutellaria baicalensis* |
| C17 | Dihydrooroxylin | 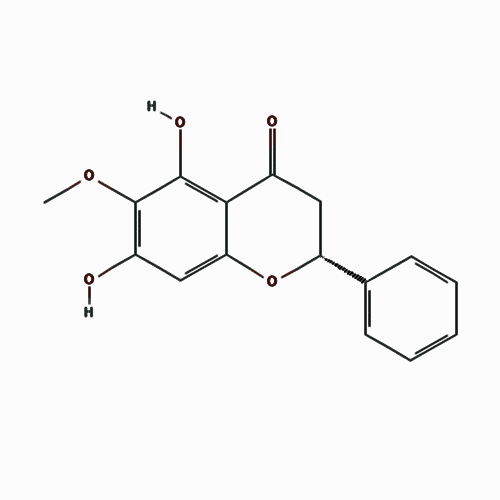 | 66.06 | 0.67 | 0.23 | *Scutellaria baicalensis* |
| C18 | Norwogonin | 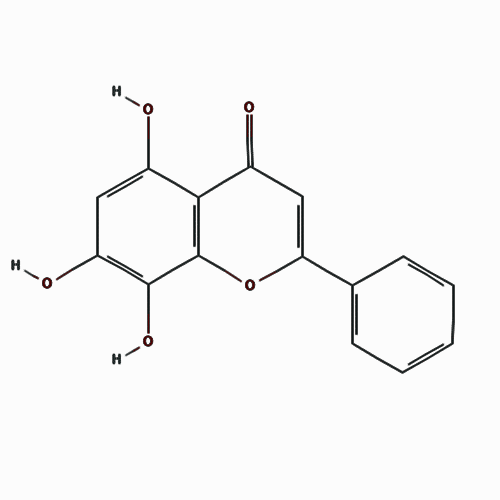 | 39.40 | 0.60 | 0.21 | *Scutellaria baicalensis* |
| C19 | Tenaxin I | 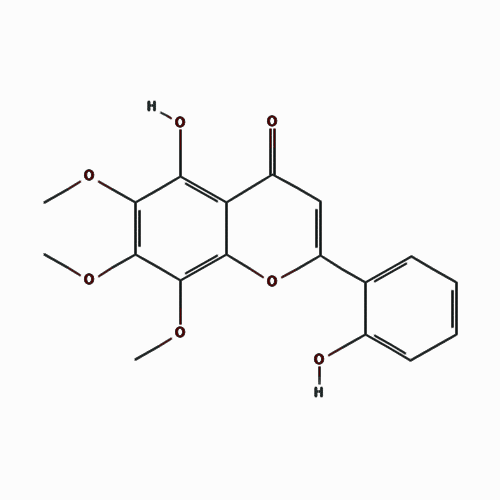 | 31.71 | 0.93 | 0.35 | *Scutellaria baicalensis* |
| C20 | Epicatechin | 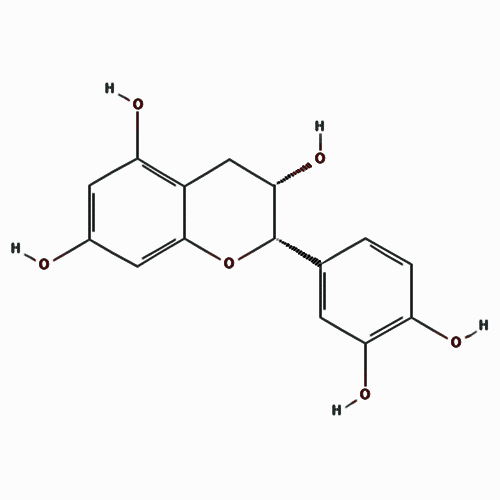 | 48.96 | 0.02 | 0.24 | *Scutellaria baicalensis* |
| C21 | Stigmasterol | 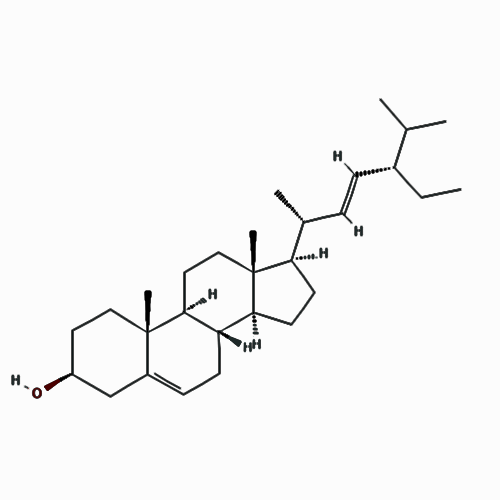 | 43.83 | 1.44 | 0.76 | *Scutellaria baicalensis* |
| C22 | Coptisine | 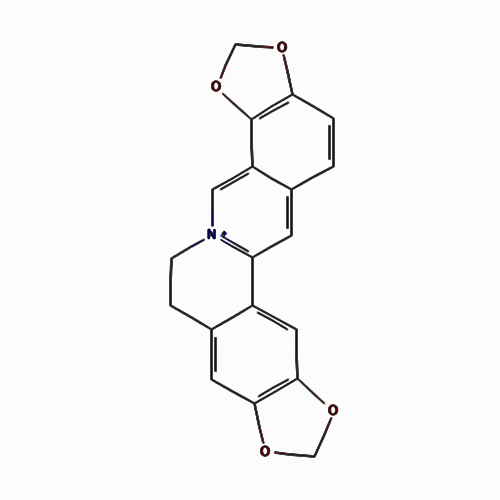 | 30.67 | 1.21 | 0.86 | *Scutellaria baicalensis* |
| C23 | Bis[(2S)-2-ethylhexyl] benzene-1,2-dicarboxylate | 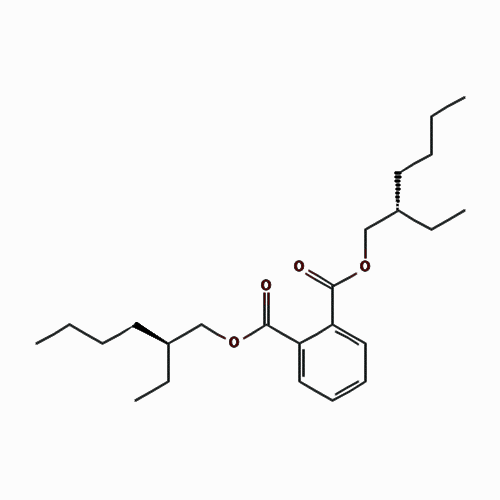 | 43.59 | 0.98 | 0.35 | *Scutellaria baicalensis* |
| C24 | Squalene | 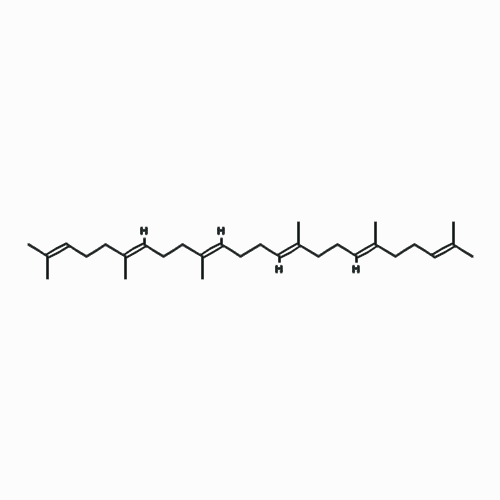 | 33.55 | 2.08 | 0.42 | *Scutellaria baicalensis* |
| C25 | Isooctyl Phthalate | 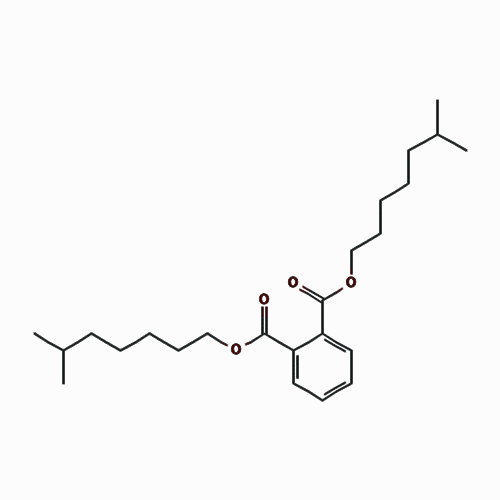 | 43.59 | 0.79 | 0.39 | *Scutellaria baicalensis* |
| C26 | Epiberberine | 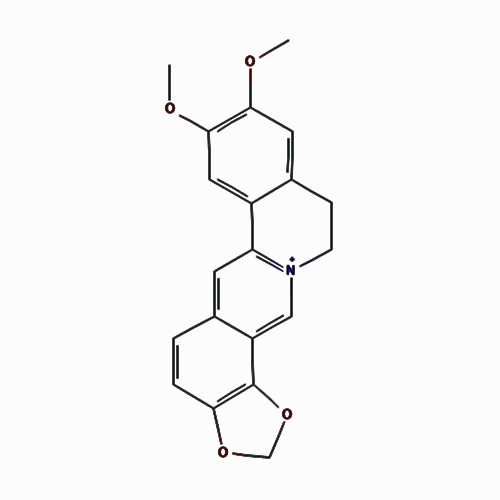 | 43.09 | 1.17 | 0.78 | *Scutellaria baicalensis* |
| C27 | 5-Hydroxy-7,8-dimethoxyflavone | 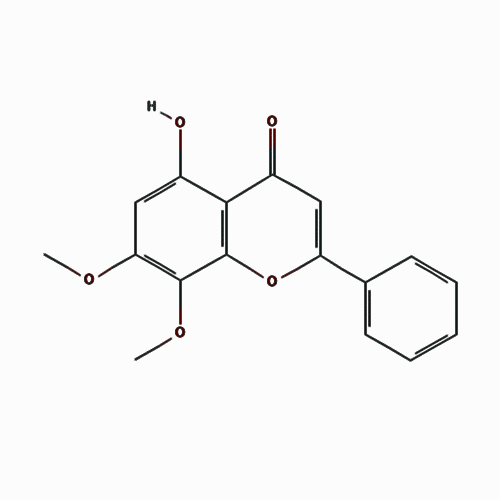 | 44.09 | 1.01 | 0.25 | *Scutellaria baicalensis* |
| C28 | Methyl 11,13-icosadienoate | 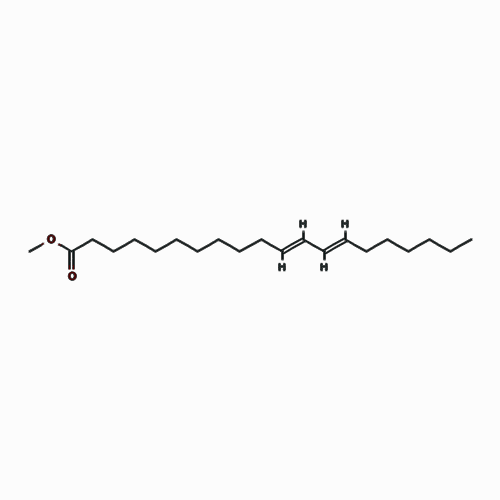 | 39.28 | 1.46 | 0.23 | *Scutellaria baicalensis* |
| C29 | 5,7,4'-Trihydroxy-6-methoxyflavanone | 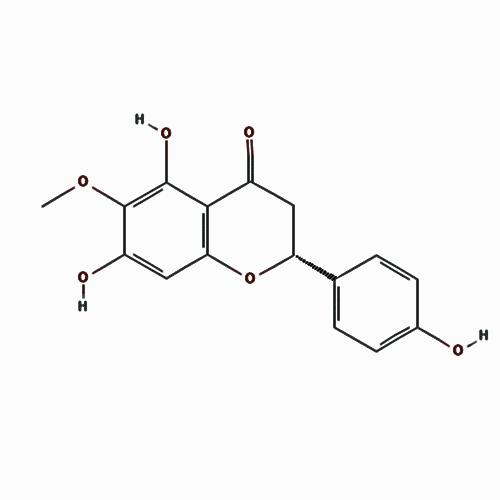 | 36.63 | 0.43 | 0.27 | *Scutellaria baicalensis* |
| C30 | 5,7,4'-Trihydroxy-8-methoxyflavanone | 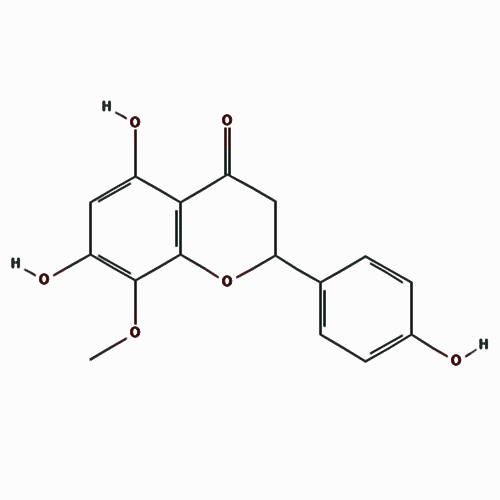 | 74.24 | 0.37 | 0.26 | *Scutellaria baicalensis* |
| C31 | Rivularin (Flavone) | 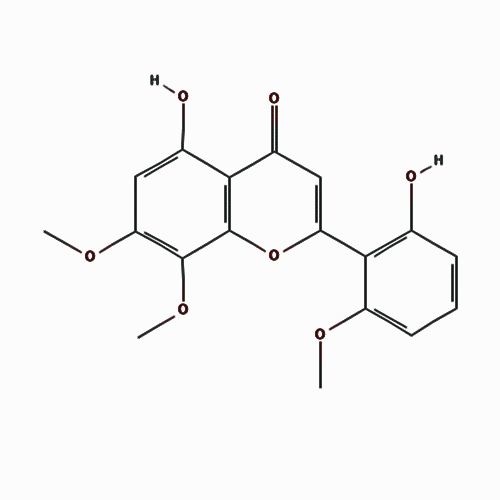 | 37.94 | 0.65 | 0.37 | *Scutellaria baicalensis* |
| C32 | Paeoniflorigenone | 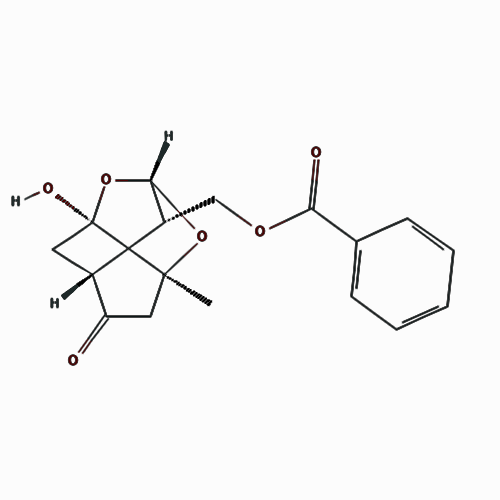 | 87.59 | -0.09 | 0.37 | *Paeonia lactiflora* |
| C33 | Palbinone | 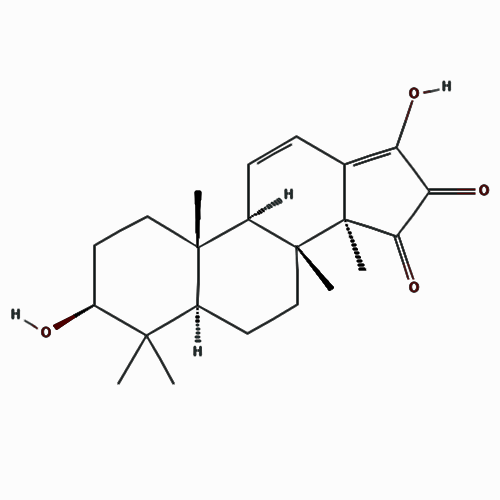 | 43.56 | 0.00 | 0.53 | *Paeonia lactiflora* |
| C34 | Betulinic Acid | 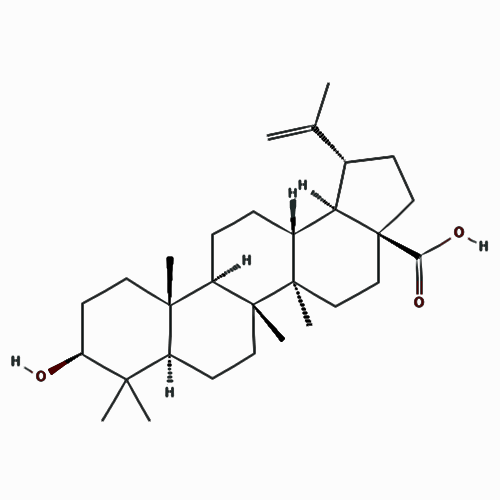 | 55.38 | 0.73 | 0.78 | *Paeonia lactiflora* |
| C35 | Kaempferol | 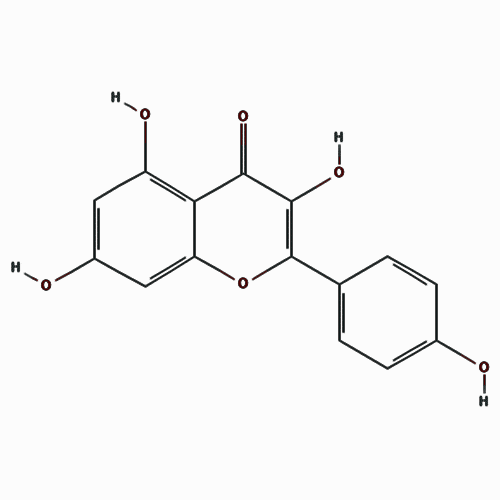 | 41.88 | 0.26 | 0.24 | *Paeonia lactiflora* |
| C36 | Cianidanol | 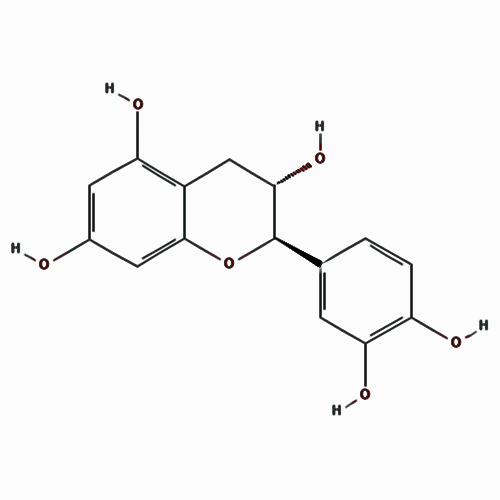 | 54.83 | -0.03 | 0.24 | *Paeonia lactiflora* |
| C37 | beta-Sitosterol | 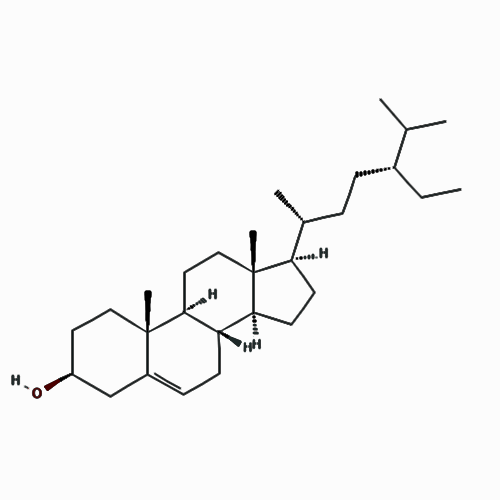 | 36.91 | 1.32 | 0.75 | *Scutellaria baicalensis & Paeonia lactiflora* |
| C38 | Sitosterol | 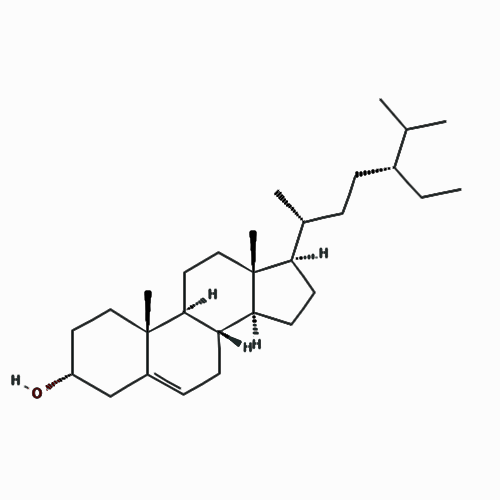 | 36.91 | 1.32 | 0.75 | *Scutellaria baicalensis & Paeonia lactiflora* |

[Supplementary](javascript:;) table 2 Targets of HQ-BS pair active compounds

| No. | Uniprot ID | Protein names | Gene names | Degree |
| --- | --- | --- | --- | --- |
| T01 | P35354 | Prostaglandin G/H synthase 2 | PTGS2 | 30 |
| T02 | P23219 | Prostaglandin G/H synthase 1 | PTGS1 | 27 |
| T03 | P07900 | Heat shock protein HSP 90-alpha | HSP90AA1 | 27 |
| T04 | P17612 | cAMP-dependent protein kinase catalytic subunit alpha | PRKACA | 21 |
| T05 | P0DP23 | Calmodulin-1 | CALM1 | 19 |
| T06 | Q15596 | Nuclear receptor coactivator 2 | NCOA2 | 19 |
| T07 | P10275 | Androgen receptor | AR | 18 |
| T08 | Q14524 | Sodium channel protein type 5 subunit alpha | SCN5A | 17 |
| T09 | P35228 | Nitric oxide synthase | NOS2 | 17 |
| T10 | P27487 | Dipeptidyl peptidase 4 | DPP4 | 17 |
| T11 | P07477 | Trypsin-1 | PRSS1 | 17 |
| T12 | P48736 | Phosphatidylinositol 4 | PIK3CG | 14 |
| T13 | P19793 | Retinoic acid receptor RXR-alpha | RXRA | 10 |
| T14 | P07550 | Beta-2 adrenergic receptor | ADRB2 | 10 |
| T15 | Q15788 | Nuclear receptor coactivator 1 | NCOA1 | 10 |
| T16 | P03372 | Estrogen receptor | ESR1 | 8 |
| T17 | Q14432 | cGMP-inhibited 3' | PDE3A | 8 |
| T18 | P35368 | Alpha-1B adrenergic receptor | ADRA1B | 8 |
| T19 | O14757 | Serine/threonine-protein kinase Chk1 | CHEK1 | 8 |
| T20 | P00734 | Prothrombin | F2 | 8 |
| T21 | P14867 | Gamma-aminobutyric acid receptor subunit alpha-1 | GABRA1 | 7 |
| T22 | P00742 | Coagulation factor X | F10 | 7 |
| T23 | P11388 | DNA topoisomerase 2-alpha | TOP2A | 7 |
| T24 | P24941 | Cyclin-dependent kinase 2 | CDK2 | 7 |
| T25 | Q12791 | Calcium-activated potassium channel subunit alpha-1 | KCNMA1 | 7 |
| T26 | Q12809 | Potassium voltage-gated channel subfamily H member 2 | KCNH2 | 7 |
| T27 | P29474 | Nitric oxide synthase | NOS3 | 7 |
| T28 | P37231 | Peroxisome proliferator-activated receptor gamma | PPARG | 7 |
| T29 | Q92731 | Estrogen receptor beta | ESR2 | 6 |
| T30 | P08709 | Coagulation factor VII | F7 | 6 |
| T31 | P10415 | Apoptosis regulator Bcl-2 | BCL2 | 6 |
| T32 | P42574 | Caspase-3 | CASP3 | 6 |
| T33 | P06401 | Progesterone receptor | PGR | 6 |
| T34 | Q07812 | Apoptosis regulator BAX | BAX | 5 |
| T35 | P20309 | Muscarinic acetylcholine receptor M3 | CHRM3 | 4 |
| T36 | P11229 | Muscarinic acetylcholine receptor M1 | CHRM1 | 4 |
| T37 | P36544 | Neuronal acetylcholine receptor subunit alpha-7 | CHRNA7 | 4 |
| T38 | P35968 | Vascular endothelial growth factor receptor 2 | KDR | 4 |
| T39 | P11217 | Glycogen phosphorylase | PYGM | 4 |
| T40 | P49841 | Glycogen synthase kinase-3 beta | GSK3B | 4 |
| T41 | Q04206 | Transcription factor p65 | RELA | 4 |
| T42 | P35348 | Alpha-1A adrenergic receptor | ADRA1A | 3 |
| T43 | P00918 | Carbonic anhydrase 2 | CA2 | 3 |
| T44 | Q16539 | Mitogen-activated protein kinase 14 | MAPK14 | 3 |
| T45 | P04637 | Cellular tumor antigen p53 | TP53 | 3 |
| T46 | P31749 | RAC-alpha serine/threonine-protein kinase | AKT1 | 3 |
| T47 | P06493 | Cyclin-dependent kinase 1 | CDK1 | 3 |
| T48 | P01857 | Immunoglobulin heavy constant gamma 1 | IGHG1 | 3 |
| T49 | P08235 | Mineralocorticoid receptor | NR3C2 | 3 |
| T50 | P08172 | Muscarinic acetylcholine receptor M2 | CHRM2 | 3 |
| T51 | P05412 | Transcription factor AP-1 | JUN | 3 |
| T52 | P21728 | D(1A) dopamine receptor | DRD1 | 2 |
| T53 | Q01959 | Sodium-dependent dopamine transporter | SLC6A3 | 2 |
| T54 | P31645 | Sodium-dependent serotonin transporter | SLC6A4 | 2 |
| T55 | P61925 | cAMP-dependent protein kinase inhibitor alpha | PKIA | 2 |
| T56 | P27338 | Amine oxidase [flavin-containing] B | MAOB | 2 |
| T57 | P18031 | Tyrosine-protein phosphatase non-receptor type 1 | PTPN1 | 2 |
| T58 | P38936 | Cyclin-dependent kinase inhibitor 1 | CDKN1A | 2 |
| T59 | Q14790 | Caspase-8 | CASP8 | 2 |
| T60 | P14635 | G2/mitotic-specific cyclin-B1 | CCNB1 | 2 |
| T61 | P35869 | Aryl hydrocarbon receptor | AHR | 2 |
| T62 | P05231 | Interleukin-6 | IL6 | 2 |
| T63 | P05177 | Cytochrome P450 1A2 | CYP1A2 | 2 |
| T64 | P22303 | Acetylcholinesterase | ACHE | 2 |
| T65 | P23975 | Sodium-dependent noradrenaline transporter | SLC6A2 | 2 |
| T66 | P28223 | 5-hydroxytryptamine receptor 2A | HTR2A | 2 |
| T67 | P34903 | Gamma-aminobutyric acid receptor subunit alpha-3 | GABRA3 | 2 |
| T68 | P55211 | Caspase-9 | CASP9 | 2 |
| T69 | P01375 | Tumor necrosis factor | TNF | 2 |
| T70 | O95433 | Activator of 90 kDa heat shock protein ATPase homolog 1 | AHSA1 | 2 |
| T71 | P03956 | Interstitial collagenase | MMP1 | 2 |
| T72 | Q92819 | Hyaluronan synthase 2 | HAS2 | 2 |
| T73 | P47869 | Gamma-aminobutyric acid receptor subunit alpha-2 | GABRA2 | 2 |
| T74 | Q03181 | Peroxisome proliferator-activated receptor delta | PPARD | 1 |
| T75 | P49327 | Fatty acid synthase | FASN | 1 |
| T76 | P48023 | Tumor necrosis factor ligand superfamily member 6 | FASLG | 1 |
| T77 | P11511 | Aromatase | CYP19A1 | 1 |
| T78 | P15692 | Vascular endothelial growth factor A | VEGFA | 1 |
| T79 | P01100 | Proto-oncogene c-Fos | FOS | 1 |
| T80 | P14780 | Matrix metalloproteinase-9 | MMP9 | 1 |
| T81 | Q16665 | Hypoxia-inducible factor 1-alpha | HIF1A | 1 |
| T82 | P15407 | Fos-related antigen 1 | FOSL1 | 1 |
| T83 | P15408 | Fos-related antigen 2 | FOSL2 | 1 |
| T84 | P05164 | Myeloperoxidase | MPO | 1 |
| T85 | P01344 | Insulin-like growth factor II | IGF2 | 1 |
| T86 | P18054 | Arachidonate 12-lipoxygenase | ALOX12 | 1 |
| T87 | O95644 | Nuclear factor of activated T-cells | NFATC1 | 1 |
| T88 | Q8NHU6 | Tudor domain-containing protein 7 | TDRD7 | 1 |
| T89 | Q9GZT9 | Egl nine homolog 1 | EGLN1 | 1 |
| T90 | Q96PH1 | NADPH oxidase 5 | NOX5 | 1 |
| T91 | Q01469 | Fatty acid-binding protein 5 | FABP5 | 1 |
| T92 | P05090 | Apolipoprotein D | APOD | 1 |
| T93 | Q9Y233 | cAMP and cAMP-inhibited cGMP 3' | PDE10A | 1 |
| T94 | P50613 | Cyclin-dependent kinase 7 | CDK7 | 1 |
| T95 | P11712 | Cytochrome P450 2C9 | CYP2C9 | 1 |
| T96 | P54289 | Voltage-dependent calcium channel subunit alpha-2/delta-1 | CACNA2D1 | 1 |
| T97 | P00326 | Alcohol dehydrogenase 1C | ADH1C | 1 |
| T98 | P08913 | Alpha-2A adrenergic receptor | ADRA2A | 1 |
| T99 | P15121 | Aldose reductase | AKR1B1 | 1 |
| T100 | P00749 | Urokinase-type plasminogen activator | PLAU | 1 |
| T101 | P09960 | Leukotriene A-4 hydrolase | LTA4H | 1 |
| T102 | P21397 | Amine oxidase [flavin-containing] A | MAOA | 1 |
| T103 | P17538 | Chymotrypsinogen B | CTRB1 | 1 |
| T104 | P08588 | Beta-1 adrenergic receptor | ADRB1 | 1 |
| T105 | P24385 | G1/S-specific cyclin-D1 | CCND1 | 1 |
| T106 | P56537 | Eukaryotic translation initiation factor 6 | EIF6 | 1 |
| T107 | Q9BXH1 | Bcl-2-binding component 3 | BBC3 | 1 |
| T108 | Q99973 | Telomerase protein component 1 | TEP1 | 1 |
| T109 | P13500 | C-C motif chemokine 2 | CCL2 | 1 |
| T110 | Q05655 | Protein kinase C delta type | PRKCD | 1 |
| T111 | P43115 | Prostaglandin E2 receptor EP3 subtype | PTGER3 | 1 |
| T112 | P02751 | Fibronectin | FN1 | 1 |
| T113 | P10145 | Interleukin-8 | CXCL8 | 1 |
| T114 | Q07820 | Induced myeloid leukemia cell differentiation protein Mcl-1 | MCL1 | 1 |
| T115 | P04040 | Catalase | CAT | 1 |
| T116 | P08173 | Muscarinic acetylcholine receptor M4 | CHRM4 | 1 |
| T117 | P31644 | Gamma-aminobutyric acid receptor subunit alpha-5 | GABRA5 | 1 |
| T118 | Q15822 | Neuronal acetylcholine receptor subunit alpha-2 | CHRNA2 | 1 |
| T119 | P35372 | Mu-type opioid receptor | OPRM1 | 1 |
| T120 | P17252 | Protein kinase C alpha type | PRKCA | 1 |
| T121 | P01137 | Transforming growth factor beta-1 proprotein | TGFB1 | 1 |
| T122 | P27169 | Serum paraoxonase/arylesterase 1 | PON1 | 1 |
| T123 | P11137 | Microtubule-associated protein 2 | MAP2 | 1 |
| T124 | O14920 | Inhibitor of nuclear factor kappa-B kinase subunit beta | IKBKB | 1 |
| T125 | P45983 | Mitogen-activated protein kinase 8 | MAPK8 | 1 |
| T126 | P47989 | Xanthine dehydrogenase/oxidase | XDH | 1 |
| T127 | P42224 | Signal transducer and activator of transcription 1-alpha/beta | STAT1 | 1 |
| T128 | P09601 | Heme oxygenase 1 | HMOX1 | 1 |
| T129 | P08684 | Cytochrome P450 3A4 | CYP3A4 | 1 |
| T130 | P04798 | Cytochrome P450 1A1 | CYP1A1 | 1 |
| T131 | P05362 | Intercellular adhesion molecule 1 | ICAM1 | 1 |
| T132 | P16581 | E-selectin | SELE | 1 |
| T133 | P19320 | Vascular cell adhesion protein 1 | VCAM1 | 1 |
| T134 | O75469 | Nuclear receptor subfamily 1 group I member 2 | NR1I2 | 1 |
| T135 | Q16678 | Cytochrome P450 1B1 | CYP1B1 | 1 |
| T136 | P09917 | Arachidonate 5-lipoxygenase | ALOX5 | 1 |
| T137 | P09211 | Glutathione S-transferase P | GSTP1 | 1 |
| T138 | O43242 | 26S proteasome non-ATPase regulatory subunit 3 | PSMD3 | 1 |
| T139 | P14672 | Solute carrier family 2 | SLC2A4 | 1 |
| T140 | Q14994 | Nuclear receptor subfamily 1 group I member 3 | NR1I3 | 1 |
| T141 | P06213 | Insulin receptor | INSR | 1 |
| T142 | P49895 | Type I iodothyronine deiodinase | DIO1 | 1 |
| T143 | Q08209 | Serine/threonine-protein phosphatase 2B catalytic subunit alpha isoform | PPP3CA | 1 |
| T144 | P09488 | Glutathione S-transferase Mu 1 | GSTM1 | 1 |
| T145 | P28161 | Glutathione S-transferase Mu 2 | GSTM2 | 1 |
| T146 | P42330 | Aldo-keto reductase family 1 member C3 | AKR1C3 | 1 |
| T147 | P03973 | Antileukoproteinase | SLPI | 1 |

Supplementary table 3 The top 20 KEGG pathways of 54 putative targets generated by DAVID

| Pathway ID | KEGG Pathway | Count of Proteins | % | *p*-Value | Class |
| --- | --- | --- | --- | --- | --- |
| hsa05200 | Pathways in cancer | 27 | 50.00 | 2.54×10^-19^ | Human Diseases; Cancers |
| hsa05161 | Hepatitis B | 19 | 35.19 | 5.18×10^-18^ | Human Diseases; Infectious diseases |
| hsa05321 | Inflammatory bowel disease (IBD) | 15 | 27.78 | 1.07×10^-17^ | Human Diseases; Immune diseases |
| hsa05144 | Malaria | 14 | 25.93 | 1.11×10^-17^ | Human diseases; Infectious diseases |
| hsa04668 | TNF signaling pathway | 17 | 31.48 | 2.35×10^-17^ | Environmental Information Processing; Membrane transport |
| hsa05164 | Influenza A | 19 | 35.19 | 1.44×10^-16^ | Human Diseases; Infectious diseases |
| hsa05142 | Chagas disease (American trypanosomiasis) | 16 | 29.63 | 4.79×10^-16^ | Human Diseases; Infectious diseases |
| hsa05323 | Rheumatoid arthritis | 15 | 27.78 | 1.25×10^-15^ | Human Diseases; Immune diseases |
| hsa05140 | Leishmaniasis | 14 | 25.93 | 2.16×10^-15^ | Human Diseases; Infectious diseases |
| hsa05145 | Toxoplasmosis | 15 | 27.78 | 3.28×10^-14^ | Human Diseases; Infectious diseases |
| hsa05205 | Proteoglycans in cancer | 18 | 33.33 | 3.45×10^-14^ | Human Diseases; Cancers |
| hsa05152 | Tuberculosis | 17 | 31.48 | 8.66×10^-14^ | Human Diseases; Infectious diseases |
| hsa04066 | HIF-1 signaling pathway | 14 | 25.93 | 1.34×10^-13^ | Environmental Information Processing; Signal transduction |
| hsa04620 | Toll-like receptor signaling pathway | 14 | 25.93 | 5.03×10^-13^ | Organismal Systems: Immune system |
| hsa05143 | African trypanosomiasis | 10 | 18.52 | 1.31×10^-12^ | Human Diseases; Infectious diseases |
| hsa05133 | Pertussis | 12 | 22.22 | 5.13×10^-12^ | Human Diseases; Infectious diseases |
| hsa05146 | Amoebiasis | 13 | 24.07 | 1.18×10^-11^ | Human Diseases; Infectious diseases |
| hsa05212 | Pancreatic cancer | 11 | 20.37 | 3.21×10^-11^ | Human Diseases; Cancers: Specific types |
| hsa05134 | Legionellosis | 10 | 18.52 | 1.60×10^-10^ | Human Diseases; Infectious diseases |
| hsa05162 | Measles | 13 | 24.07 | 1.77×10^-10^ | Human Diseases; Infectious diseases |
