# Supplementary figures and images for: Herbal pair Huangqin-Baishao: mechanisms underlying inflammatory bowel disease by combined system pharmacology and cell experiment approach
Source: BMC Complement Med Ther. 2020 Sep 25;20:292. doi: 10.1186/s12906-020-03068-2 (PMC7523401; doi:10.1186/s12906-020-03068-2)

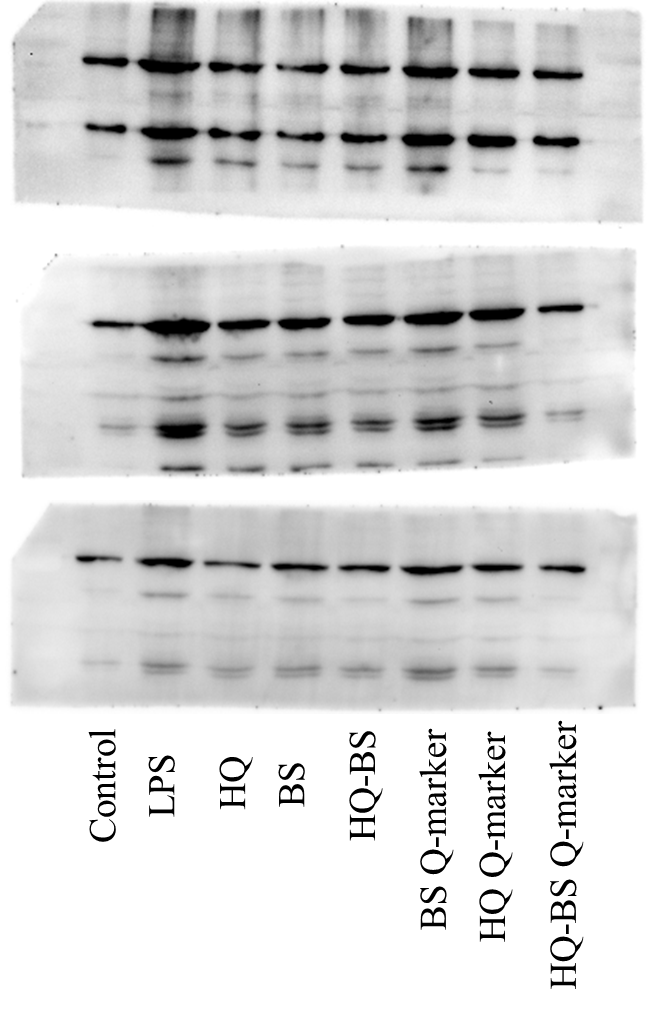


Figure 10 iNOS blots


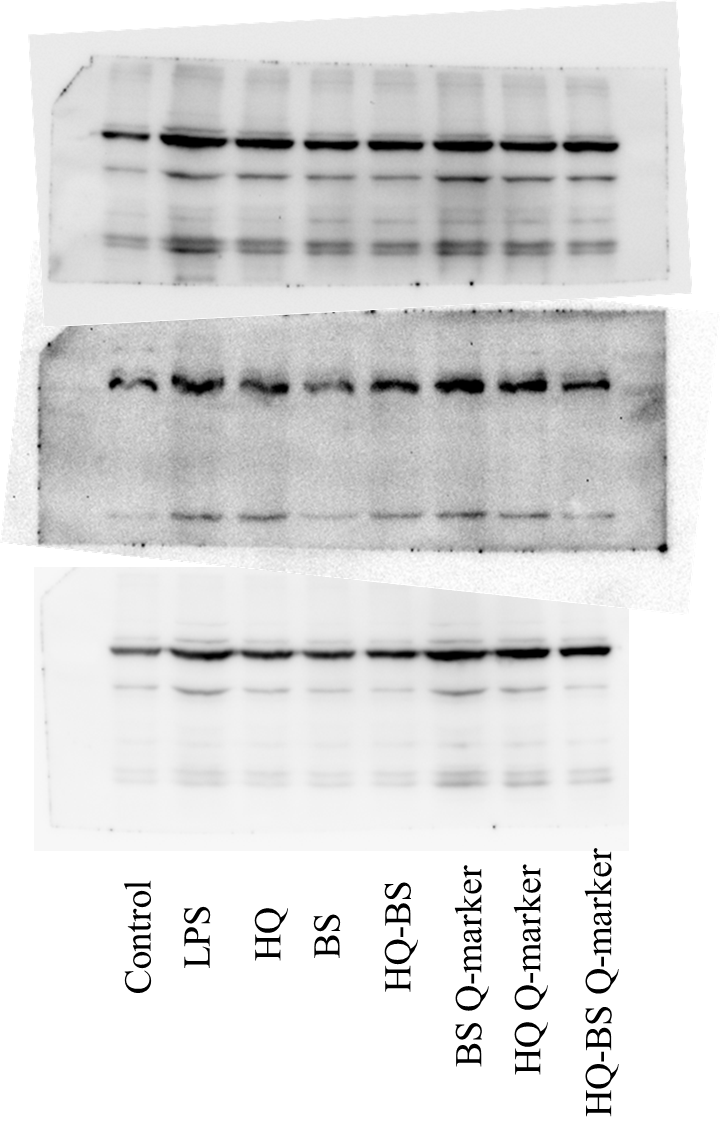


Figure 10 COX-2 blots


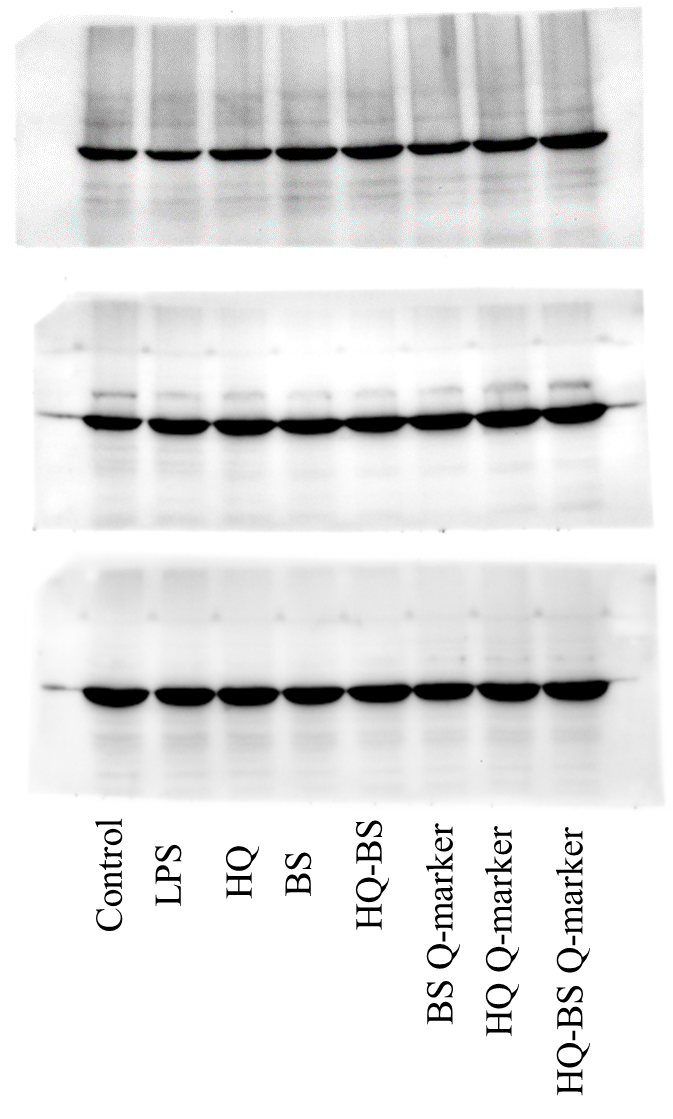


Figure 10 β-actin blots

Supplement: Supplementary file 2 — Additional file 2. Original blot images. [file 12906_2020_3068_MOESM2_ESM.docx]
